# Appraisal of selected ethnomedicinal plants as alternative therapies against onychomycosis: Evaluation of synergy and time-kill kinetics

**DOI:** 10.3389/fphar.2022.1067697

**Published:** 2022-11-24

**Authors:** Syeda Aroosa Mohsin, Shazia Shaukat, Marya Nawaz, Tofeeq Ur-Rehman, Nadeem Irshad, Muhammad Majid, Syed Shams ul Hassan, Simona Bungau, Humaira Fatima

**Affiliations:** ^1^ Department of Pharmacy, Faculty of Biological Sciences, Quaid-i-Azam University, Islamabad, Pakistan; ^2^ Department of Pathology, Shifa College of Medicine, Islamabad, Pakistan; ^3^ Faculty of Pharmacy, Hamdard University, Islamabad, Pakistan; ^4^ Shanghai Key Laboratory for Molecular Engineering of Chiral Drugs, School of Pharmacy, Shanghai Jiao Tong University, Shanghai, China; ^5^ Department of Natural Product Chemistry, School of Pharmacy, Shanghai Jiao Tong University, Shanghai, China; ^6^ Department of Pharmacy, Faculty of Medicine and Pharmacy, University of Oradea, Oradea, Romania

**Keywords:** onychomycosis, antifungal, resistance, synergistic studies, HPLC

## Abstract

**Introduction:** This study aims at the biological profiling of *Allium sativum*, *Zingiber officinale*, *Nigella sativa*, *Curcuma longa*, *Mentha piperita*, *Withania somnifera*, *Azadirachta indica*, and *Lawsonia inermis* as alternatives against onychomycosis to combat the treatment challenges.

**Methods:** An extract library of aqueous (DW), ethyl acetate (EA), and methanol (M) extracts was subjected to phytochemical and antioxidant colorimetric assays to gauge the ameliorating role of extracts against oxidative stress. RP-HPLC quantified therapeutically significant polyphenols. Antifungal potential (disc diffusion and broth dilution) against filamentous (dermatophytes and non-dermatophytes) and non-filamentous fungi (*yeasts*; *Candida albicans*), synergistic interactions (checkerboard method) with terbinafine and amphotericin-B against resistant clinical isolates of dermatophytes (*Trichophyton rubrum* and *Trichophyton tonsurans*) and non-dermatophytes (*Aspergillus* spp., *Fusarium dimerum*, and *Rhizopus arrhizus*)*,* time-kill kinetics, and protein estimation (Bradford method) were performed to evaluate the potential of extracts against onychomycosis.

**Results:** The highest total phenolic and flavonoid content along with noteworthy antioxidant capacity, reducing power, and a substantial radical scavenging activity was recorded for the extracts of *Z. officinale*. Significant polyphenolics quantified by RP-HPLC included rutin (35.71 ± 0.23 µg/mgE), gallic acid (50.17 ± 0.22 µg/mgE), catechin (93.04 ± 0.43 µg/mgE), syringic acid (55.63 ± 0.35 µg/mgE), emodin (246.32 ± 0.44 µg/mgE), luteolin (78.43 ± 0.18 µg/mgE), myricetin (29.44 ± 0.13 µg/mgE), and quercetin (97.45 ± 0.22 µg/mgE). Extracts presented prominent antifungal activity against dermatophytes and non-dermatophytes (MIC-31.25 μg/ml). The checkerboard method showed synergism with 4- and 8-fold reductions in the MICs of *A. sativum*, *Z. officinale*, *M. piperita*, *L. inermis*, and *C. longa* extracts and doses of amphotericin-B (Amp-B) and terbinafine (against non-dermatophytes and dermatophytes, respectively). Furthermore, the synergistic therapy showed a time-dependent decrease in fungal growth even after 9 and 12 h of treatment. The inhibition of fungal proteins was also observed to be higher with the treatment of synergistic combinations than with the extracts alone, along with the cell membrane damage caused by terbinafine and amp-B, thus making the resistant fungi incapable of subsisting.

**Conclusion:** The extracts of *A. sativum*, *Z. officinale*, *M. piperita*, *L. inermis*, and *C. longa* have proven to be promising alternatives to combat oxidative stress, resistance, and other treatment challenges of onychomycosis.

## 1 Introduction

Onychomycosis, a fungal nail infection accounting for almost 50% of all nail disorders, is chiefly characterized by hyperkeratosis and nail discoloration, leading to brittleness and periungual inflammation^1^ (Piraccini and Alessandrini, 2015) (Shi et al., 2016). It is caused by dermatophytes (*Trichophyton rubrum*, and *T. tonsurans*), non-dermatophyte molds (*Aspergillus* spp., *Fusarium* spp., and *Rhizopus* spp.), and yeasts (*Candida* spp.) (Farwa et al., 2011). Dermatophytes are keratinophilic fungi that spread quickly and invade keratin-rich substrates. They obtain their nutrients from the nail as a result. Some species also live as saprobes and obtain their sustenance from soil-found bits of feathers, nails, and hair. Without creating symptoms or lesions in the integumentary tissue, the anthropophilic ones can colonize within people. The disulfide bonds of the amino acid l-cysteine in keratin are thought to be cleaved during the keratinolytic process using sulfite generated by dermatophytes (de Souza Costa et al., 2022). In cases of severe immunosuppression, yeasts mostly damage the nails. As one of their many virulence mechanisms, extracellular hydrolases (such as proteinases, phospholipases, and lipases) produced by yeasts can contribute to the pathogenesis of nail infections (Leitão, 2020). Non-dermatophytes such as *Fusarium* spp. and *Aspergillus* spp., although, are not classified as keratolytics, and some of these (e.g., *Fusarium* spp.) have the ability to degrade keratin and use the nail as a nutritional source, causing the infection. These produce toxins and exocellular products that can permeate the skin and induce an inflammatory response and apoptosis. In addition, these induce the production of pro-inflammatory cytokines, causing dermal changes in the production of extracellular matrix (ECM). This process is expected to be responsible for paronychia, which is frequent in onychomycosis (Veiga et al., 2018) (Guilhermetti et al., 2007) (Correia et al., 2020). Thiol/disulfide homeostasis is indicative of oxidative stress. Onychomycosis patients revealed a thiol/disulfide homeostasis shift to oxidative stress with thiol reduction and a rise in the disulfide/native thiol, and disulfide/total thiol ratios. These outcomes may suggest the role of oxidative stress in the pathogenesis of onychomycosis (Metin et al., 2021). Several types of onychomycosis entail total dystrophic onychomycosis, white superficial onychomycosis, distal and subungual onychomycosis, endonyx onychomycosis, and proximal onychomycosis (Aggarwal et al., 2020). Onychomycosis, if untreated, may affect the life quality, prevalence, and severity of foot ulcers in patients with a compromised immune system, diabetes mellitus, or peripheral arterial disease (Malik and Raza, 2009). Therefore, therapeutic strategies for onychomycosis should include immunomodulation/stimulation, reduction of inflammation, and oxidative stress. Current therapy for onychomycosis comprises both oral (terbinafine, fluconazole, and itraconazole) and topical (terbinafine, efinaconazole, amorolfine, and ciclopirox) antifungals (ShariLipnerMD, 2019). However, poor drug penetration owing to hyperkeratosis in case of topical application, resistance to contemporary antifungals, particularly of Trichophyton species against terbinafine, prolonged duration of therapy, drug–drug interactions, and adverse effects are some of the clinical challenges in treating the disease (Thampi et al., 2022; Falotico and Lipner, 2022).

Plants contain a plethora of secondary metabolites of diverse chemical structures such as terpenoids, tannins, saponins, flavonoids, and alkaloids (Mahmood et al., 2017; Ayaz et al., 2015) that have exhibited multiple pharmacological activities, including anti-cancer (Hassan et al., 2022a), anti-inflammatory (Muhammad et al., 2021), anti-diabetic, immunomodulatory, anti-bacterial (Majid et al., 2022), and antifungal activities (Ayaz et al., 2016; Khalil et al., 2022). The successful history of natural moieties in antifungal drug discovery requires no further advocacy as two out of three approved antifungal drug classes, i.e., polyenes and echinocandins, are natural products or natural product-derived and therefore represent a novel strategy to enrich our antifungal arsenal (Ovais et al., 2018). Recently, several scientific studies have shown that contemporary antifungal agents that have become resistant could re-sensitize the pathogen when incorporated in combination with phytochemicals, even at lower concentrations, than their reported minimum inhibitory concentration (MIC) (Hassan et al., 2017). Therefore, in the undertaken study, a panel of plants such as *Allium sativum*, *Zingiber officinale*, *Nigella sativa*, *Curcuma longa*, *Mentha piperita*, *Withania somnifera*, *Azadirachta indica*, and *Lawsonia inermis*, (plant names have been checked with http://www.theplantlist.org), with a history of traditional medicinal uses as antiseptics, antimicrobials, or against skin disorders (Table 2), was selected to unleash their potential against onychomycosis. HPLC-based quantification of therapeutically significant polyphenols and the oxidative stress-ameliorating potential of extracts using a battery of antioxidant assays were determined to extrapolate their possible roles in the treatment of onychomycosis. To the best of our knowledge, the determination of synergism between contemporary antifungals (terbinafine and amphotericin B) and an extract library of selected plants using a checkerboard platform and the mechanistic insight into the observed activity (by establishing time-kill kinetic studies and total protein content estimation) have not been described hitherto.

## 2 Materials and methods

### 2.1 Chemicals and reagents

Distilled water (DW), dimethyl sulfoxide (DMSO), methanol (MeOH), ethyl acetate (EA), standard antifungals (amphotericin B and terbinafine), nutrient agar, and HPLC-grade polyphenols (vanillic acid, rutin, plumbagin, thymoquinone, gallic acid, catechin, syringic acid, coumaric acid, emodin, gentisic acid, caffeic acid, ferulic acid, luteolin, apigenin, myricetin, quercetin and kaempferol), 2,2-diphenyl-1-picryhydrazyl (DPPH), ascorbic acid, sulfuric acid, gallic acid, potassium acetate, quercetin, ammonium molybdate, aluminum chloride, trichloroacetic acid (TCA), potassium ferricyanide, and ferric chloride were purchased from Sigma Aldrich, United States. Phosphate buffer and Folin–Ciocalteu (FC) reagent were procured from Riedel-de Haen, Germany. Sabouraud dextrose agar (SDA) was purchased from Oxoid, England, Tween-80 from Merck-Schuchardt, United States, and RPMI-1640 was obtained from United traders, Rawalpindi, Pakistan.

### 2.2 Cultures and strains

Non-dermatophyte fungal strains included in the current study were *Aspergillus niger* (FCBP-0198), *Aspergillus flavus* (FCBP 0064), and *Mucor* spp. (FCBP 0300). Clinical isolates of non-dermatophytes, including *Aspergillus terreus*, *Rhizopus arrhizus*, *Fusarium dimerum*, *Alternaria alternata,* and dermatophytes. including *Trichophyton rubrum* and *Trichophyton tonsurans,* were provided by the Armed Forces Institute of Pathology (AFIP), Combined Military Hospital (CMH), Rawalpindi. Among yeasts, *Candida albicans* (ATCC 10231) was used for screening.

### 2.3 Plant materials and preparation of crude extracts

The selected plants (parts used for extraction have been mentioned in Table 2) were collected from the local healers (Hakims) of Rawalpindi, Pakistan and authenticated by Prof. Dr. Rizwana Aleem Qureshi, Department of Plant Sciences, Faculty of Biological Sciences, Quaid-i-Azam University Islamabad, Pakistan, and the plant specimens were added under voucher numbers (*Allium sativum*-PHM 562), (*Zingiber officinale*-PHM 569), (*Nigella sativa*-PHM 567), (*Curcuma longa*-PHM 564)*,* (*Mentha piperita*-PHM 566), (*Withania somnifera*-PHM 568)*,* (*Azadirachta indica*-PHM 563), and (*Lawsonia inermis*-PHM 565) within the herbarium of medicinal plants, Department of Pharmacy, Quaid-i-Azam University, Islamabad for further reference. Exhaustive solvent extraction with ultra-sonication-aided maceration using three analytical-grade solvents—ethyl acetate (EA), methanol (MeOH), and distilled water (DW)—was performed to prepare the extracts. 500 g of each selected plant was subjected to maceration for 24 h at room temperature, followed by periodic sonication. The marc was extracted twice using the same procedure, and the filtrates were combined, followed by filtration through a muslin cloth and finally through Whatmann No. 1 filter paper. The extracts were concentrated in a rotary evaporator (Buchi, Switzerland) through vacuum evaporation and ultimately dried in a vacuum oven (Yamato, Japan) at 45°C to obtain the final crude extracts (Fatima et al., 2015). Extracts after proper labeling were stored at 4°C in the refrigerator until further use.

### 2.4 Antioxidant assays

#### 2.4.1 DPPH free radical scavenging assay

The antioxidant potential of the tested extracts was estimated by their capacity to scavenge the stable 2, 2-diphenyl 1-picrylhydrazyl (DPPH) free radical (Fatima et al., 2015). From each sample, 10 µl was added to the respective well, followed by addition of 190 µl of DPPH stock. The mixture was incubated in the dark at 37°C for 60 min. The absorbance of the samples was measured at 515 nm with a microplate reader (Elx 800, Biotek, United States). The following formula was used to calculate the percent of scavenging activity:

$$\%\;scavenging\;activity=\left(1-\frac{Abs}{Abc}\right)\times 100,$$

where, Abs, absorbance of the sample; Abc, absorbance of the control.

The whole assay was run in triplicate. Ascorbic acid was employed as a positive control and DMSO as a negative control. The tested extracts presenting more than 50% scavenging activity at an initial concentration of 200 μg/ml were further put to test for calculation of IC_50_ using three-fold serial dilutions (Ul Hassan et al., 2021).

#### 2.4.2 Total antioxidant capacity assay

The total antioxidant capacity of the tested extracts was determined as previously described (Fatima et al., 2015). In an Eppendorf tube, 100 µl of the stock solution of samples (4 mg/ml) was taken with subsequent addition of 900 µl of TAC reagent. The mixture was incubated in a water bath at 95°C for 90 min and then cooled at room temperature. Ascorbic acid and DMSO were used as positive and negative controls, respectively, and the whole procedure was repeated three times. The absorbance was recorded at 630 nm. The antioxidant capacity of the tested extracts was expressed as microgram ascorbic acid equivalent of plant extract (μg AAE/mgE).

#### 2.4.3 Total reducing power assay

The total reducing power by potassium ferricyanide colorimetric assay formerly reported (Fatima et al., 2015) was utilized. First, in Eppendorf tubes, 100 µl from each sample stock was added, followed by the addition of phosphate buffer, 200 µl (0.2 M) and then from 1% potassium ferricyanide [K_3_Fe (CN)_6_], 250 µl was added on. The Eppendorf tubes containing the sample mixture were incubated for 20 min at 50°C and afterward, 200 μl of 10% trichloroacetic acid was added. This mixture was then centrifuged at room temperature for 10 min at 3,000 rpm. In the meantime, 50 μl ferric chloride (FeCl_3_) was added to each well of 96-well plates. After centrifugation, from the supernatant, an aliquot of 150 μl was transferred to each corresponding well, and the absorbance of samples at 593 nm was measured. 1 mg/ml ascorbic acid at various concentrations of 25, 12.5, 6.25, and 3.125 μg/ml was used as a positive control, while DMSO was used as a negative control for the assay. The whole assay was performed in triplicate analysis, and the reducing power of the tested extracts was expressed as microgram of ascorbic acid equivalent of extract (μg AAE/mgE).

### 2.5 Phytochemical analysis

#### 2.5.1 Determination of total phenolic content

The total phenolic content of the tested extracts was determined using the Folin–Ciocalteu (FC) reagent according to the previously reported procedure (Fatima et al., 2015). In each corresponding well of a 96-well plate, an aliquot of 20 µl was added from the 4 mg/ml stock solution of each extract, followed by the addition of 90 µl of FC reagent. The plate was incubated at 37°C for 30 min, and afterward, 90 µl of sodium bicarbonate was added to each well. The absorption of the sample extracts was recorded at 630 nm using a microplate reader. Gallic acid in two-fold serial dilutions (2.5, 5, 10, 20, and 40 μg/ml) was used as a positive control to obtain the calibration curve y = 0.0276x + 0.0034 (*R* ^2^ = 0.9991), and DMSO was employed as a negative control. The results of this assay were expressed as microgram gallic acid equivalent of the extract (μg AAE/mgE), and the procedure was performed in triplicate.

#### 2.5.2 Determination of total flavonoid content

For total flavonoid content estimation, the aluminum chloride calorimetric method was employed (Jafri et al., 2017). From the tested extracts, an aliquot of 20 µl was transferred to each well, following which 10 µl potassium acetate, aluminum chloride (10 µl), and 160 µl of distilled water was added to the respective wells. The plate was incubated for 30 min at room temperature, and the absorption was measured at 415 nm with the aid of a microplate reader. The positive control was quercetin at different concentrations (0, 2.5, 5, 10, 20, and 40 μg/ml), while DMSO was employed as the negative control. The calibration curve of quercetin obtained was y = 0.0631x − 0.0112 (*R* ^2^ = 0.9988). The assay was performed thrice, and the results were presented as microgram quercetin equivalent of the extract (μg AAE/mgE).

#### 2.5.3 Reverse phase-high-performance liquid chromatography

For HPLC to be performed, Agilent Chem Station Rev. B.02-01-SR1 (260) and an Agilent 1,200 series binary gradient pump coupled with a multiple wavelength detector (MWD) were used. A Zorbex-C8 analytical column (4.6 mm × 250 mm, 5 μm particle size), gradient elution, and injection volume of 50 μl were selected to perform reverse phase chromatographic analysis as previously reported (Fatima et al., 2015). The mobile phase consisted of solvent A: acetonitrile-methanol–water-acetic acid (5:10:85:1) and solvent B: acetonitrile–methanol–acetic acid (40:60:1). The gradient method for 0%–50% was 0–20 min, for 50–100% B was 20–25 min, and then isocratic until 30 min for 100% B. The flow rate was kept at 1 ml/min. HPLC-grade methanol was used to prepare stock solutions of reference phenolic standards (vanillic acid, rutin, plumbagin, thymoquinone, gallic acid, catechin, syringic acid, coumaric acid, emodin, gentisic acid, caffeic acid, ferulic acid, luteolin, apigenin, myricetin, quercetin, and kaempferol) to obtain final concentrations of 5, 10, 20, 50 and 100 μg/ml of methanol. A calibration curve was calculated using the data for peak area *versus* the standard concentration. Standard solutions, mobile phases, and samples were degassed prior to use and filtered through a 0.45-μm membrane filter. The wavelengths for absorption of samples were 257 nm (vanillic acid, rutin, plumbagin, and thymoquinone), 279 nm (gallic acid, catechin, syringic acid, coumaric acid, and emodin), 325 nm (gentisic acid, caffeic acid, ferulic acid, luteolin, and apigenin), and 368 nm (myricetin, quercetin, and kaempferol).

A comparison of the retention time and UV absorption spectra of extracts with standards was carried out for the identification of compounds, and the results were expressed as microgram/milligram of the extract. The chromatographic procedure was performed at ambient temperature and in triplicate. Prior to the next analysis, the column was reconditioned for 10 min.

### 2.6 Preliminary resistance profiling of antifungals

Initially, antifungal drugs were tested against clinical isolates of dermatophytes and non-dermatophytes by the broth dilution method (Wayne, 2008). Stock solutions (4 mg/ml) of antifungals (terbinafine and amphotericin-B) were prepared in DMSO. RPMI-1640 was used as a culture medium. The turbidity of the inoculum (in sterile saline, 0.85%) was adjusted according to McFarland standard 0.5. 5 µl from each antifungal stock and 95 µl of RPMI were transferred into the corresponding wells of the microtiter plate, after which 100 µl of the fungal suspension of the respective strain was added in each well. For dermatophytes, terbinafine was used, and for non-dermatophytes, amphotericin B was used as a positive control, and DMSO diluted in RPMI-1640 (less than 1%) was used as a negative/growth control. The plates were incubated at 35°C for periods varying according to strains or till growth appeared in the growth control well. The results were recorded with a visual aid using a magnifying mirror. The MIC was expressed as the last well, which was clear of any growth. The assay was performed three times.

### 2.7 Antifungal susceptibility testing

For the estimation of *in vitro* antifungal activity of crude extracts, microbroth dilution was employed. The protocol devised by the Clinical and Laboratory Standards Institute (CLSI) in document M38-A2 was followed (Wayne, 2008). RPMI-1640 was used as a culture medium to sustain fungal growth. 0.85% sterile saline was used for inoculum preparation. Spores were transferred through a Pasteur pipette to a saline solution containing Tween 20. The turbidity was adjusted according to the McFarland 0.5 standard. 5 µl from each sample stock was transferred into the corresponding wells of a microtiter plate, after which 195 µl of RPMI was added to all respective wells (500 μg/ml RPMI). Then, two-fold serial dilutions containing 250 μg/ml, 125 μg/ml, 62.5 μg/ml, and 31.25 μg/ml RPMI were prepared in the next wells. After sample addition, 100 µl fungal suspension of the respective strain was added to each well. For dermatophytes, terbinafine at a final concentration ranging from 0.5–0.001 μg/ml RPMI and for non-dermatophytes, amphotericin B (16–0.313 μg/ml RPMI) was used as a positive control, and DMSO diluted in RPMI-1640 (less than 1%) was used as a negative/growth control. The plates were incubated at 35°C for periods varying according to strains or till growth appeared in the growth control well. The results were recorded with a visual aid using a magnifying mirror. The MIC was expressed as the last well, which is clear of any growth. The whole assay was run in triplicate.

For screening against *Candida albicans*, the disc diffusion method was employed, as previously explicated (Bakht et al., 2014). Concisely, 20 ml of SDA was poured into Petri plates, and spores were spread on the agar surface. Filter paper discs were used for impregnation of samples. Sample extracts with a final concentration of 100 μg/ml DMSO on each disc were placed on the inoculated agar plates. Amphotericin B was used as a positive control, and DMSO was used as negative control. The plates were then incubated for a period of 4 days at 28°C or until the growth appeared. Zones of inhibition (ZOI) were measured using a Vernier caliper. The assay was performed in triplicate analysis.

### 2.8 Determination of synergy

For the estimation of interactions between plant extracts and contemporary antifungal agents (terbinafine for dermatophytes and amphotericin B for non-dermatophytes), the checkerboard method, as previously described (Bidaud et al., 2021), was used in accordance with CLSI guidelines (document M38-A2) (Wayne, 2008). Sample extracts were added to 96-well plates horizontally, while terbinafine was added vertically in various concentrations. The first concentration of each sample extract was 62.5 μg/ml RPMI (2MIC), and from this concentration, two-fold serial dilutions were prepared. The different concentrations of extracts included 62.5, 31.25, 15.625, 7.8125, and 3.906 μg/ml RPMI. For terbinafine, the final concentrations included in the assay were 0.01 (2MIC), 0.005, 0.0025, 0.00125, and 0.000625 μg/ml RPMI. For amphotericin-B, different concentrations used in the assay included 6.4 (2MIC), 3.2, 1.6, 0.8, and 0.4 μg/ml RPMI. For addition of both antifungals in a well, 50 µl from the first concentrations of both extracts and terbinafine/amphotericin-B was transferred in a well, followed by the addition of 100 µl inoculum. The same procedure was followed for the next respective concentrations of extracts, terbinafine and amphotericin B. The positive control for dermatophytes was terbinafine, and for non-dermatophytes, amphotericin B was employed, while less than 1% DMSO was used as a negative control for the assay (Cuenca-Estrella, 2004), (Iten et al., 2009). The whole procedure was repeated in triplicate. The fractional inhibitory index (FIC) was calculated using the following formula:

FIC of antifungal=MIC Of antifungal in combination/ MIC of antifungal alone,

FIC of extract (FIC extract)=MIC of extract in combination/ MIC of extract alone,

while the FIC index was the sum of the FIC of both antifungal and plant extract,

FIC Index (FICI)=FICantifungal+FICextract

FICI values are interpreted as indicated in Table 1.

**TABLE 1:** Interpretation of FICI values.

FICI values	Interpretation
≤0.5	Synergistic
>4	Antagonistic
>0.5–1	Additive
>1 and <4	Indifferent

### 2.9 Time-kill kinetics

Fungi were grown to mid-logarithmic phase, and then the diluted fungal suspension (10^4^ CFU/ml) was incubated with MIC, 2MIC, FICI, and 2FICI of extracts and their combinations at 35°C for 12 h. Absorbance was recorded at 0, 3, 6, 9, and 12 h at 600 nm. Fungal growth was observed by plotting a graph between absorbance and time accordingly (Selestino Neta et al., 2017). The experiment was run in triplicate.

### 2.10 Protein estimation assay

For the estimation of protein content in fungi alone, treated with extract, and those treated with extracts and antifungal drugs, it was determined using the Bradford method as formerly described (Nouroozi et al., 2015; Li et al., 2019; Qu et al., 2020). Protein standard consisted of bovine serum albumin (BSA, A8806 Sigma) at 1 mg/ml prepared in phosphate buffer. Different concentrations of the protein standard (0–50 μg/ml) diluted in phosphate buffer were used as positive controls. The assay was performed using 5 µl of each test sample, after which 195 µl of Bradford reagent was added to microplate wells. The content was mixed continuously for 60 s. The plate was incubated at room temperature for 5 min, and absorbance was measured at 595 nm using a microplate reader (Elx 800, Biotek, United States). The color of samples was observed after adding the reagent, and a change to a blue or purple–blue color was obtained if protein content was present in the solution. The standard curve and calibration equation y = 0.0104x − 0.0016 (*R* ^2^ = 0.9992) were generated by plotting the absorbance of standard dilutions *versus* their concentrations. The protein concentration of the sample was calculated using the following formula:

y=mx+b.

Here *x* is the unknown concentration of the protein, y is absorbance, b is the intercept, and m is the slope of the standard curve.

## 3 Statistical analysis

The data were presented as the mean of triplicates ± standard error. Graph Pad Prism software 5.0 was applied for graphical representations and for IC_50_ calculations.

## 4 Results and discussion

In onychomycosis, most infections by dermatophytes are caused by *Trichophyton rubrum* and *T. mentagrophytes,* while in yeast, the most common causative species include *Candida* spp. (Mügge et al., 2006). The non-dermatophyte species that cause onychomycosis are *Aspergillus*, *Fusarium*, *Alternaria*, *Cladosporium*, *Acremonium*, and *Scopulariopsis* (Farwa et al., 2011; Zou et al., 2022).

Since the plants are rich in secondary products such as flavonoids, saponins, tannins, terpenoids, and alkaloids, several plants have been shown to have antifungal activity or have served as a guide for discovery of novel anti-fungal drugs. Various plant compounds, such as dimethylpyrrole, indole derivatives, and hydroxyhydrochornylcones, are stated to possess antifungal effects (Hassan et al., 2017). In the present study, a panel of ethnomedicinal plants with traditional use as antiseptics, antimicrobials, or for treatment of skin infections was selected to appraise their potential use against onychomycosis (Table 2).

**TABLE 2:** Selected plants for antifungal screening.

Biological source	Vernacular names	Part(s) used for extraction	Family	Ethnomedicinal uses	References
*Allium sativum*	Garlic	Cloves	Amaryllidaceae	Reducing blood pressure, cholesterol levels, and headache, as an antiseptic, anti-inflammatory, and prevention of cancer	Alam et al. (2016)
*Azadirachta indica*	Neem	Leaves	Meliaceae	Antimalarial, anti-pyretic, and antiseptic, in skin infections such as eczema, antidiabetic, anticancer, and anthelmintic	Hashmat et al. (2012)
*Curcuma longa*	Turmeric	Rhizomes	Zingiberaceae	Antiseptic, as a disinfectant, anti-inflammatory, to treat skin infections, analgesic also aids in digestion	Verma et al. (2018)
*Lawsonia inermis*	Henna	Leaves	Lythraceae	Headache, skin problems, amebiasis, protective against lice and dandruff, enlargement of the spleen, jaundice, treating ulcers, and in digestive problems	(Al-Snafi, 2019), (Semwal et al., 2014)
*Mentha piperita*	Mint	Leaves	Lamiaceae	Analgesic, treating cold, digestive problems, throat inflammation, antiviral, and antifungal	Mahendran and Rahman, (2020)
*Nigella sativa*	Black seeds	Fruit	Ranunculaceae	Anti-hypertensive, liver tonic, antidiarrheal, diuretic, appetite-stimulant, antidiabetic, antibacterial, analgesic, and in skin infections	(Majeed et al., 2020), (Ahmad et al., 2013)
*Withania somnifera*	Ashwagandha	Seeds	Solanaceae	Anti-inflammatory, treating ulcers, conjunctivitis, cold and coughs, anti-epileptic, leprosy, insomnia, anti-arthritic, asthma, and intestinal diseases	Umadevi et al. (2012)
*Zingiber officinale*	Ginger	Rhizomes	Zingiberaceae	Treating heart complications, food poisoning, osteo-arthritis, menstruation disorders, anti-epileptic, nausea, cough, inflammation, cancer, and travel sickness	Kumar Gupta and Sharma, (2014)

### 4.1 Antioxidant assays

#### 4.1.1 DPPH free radical scavenging

**FIGURE 1:**
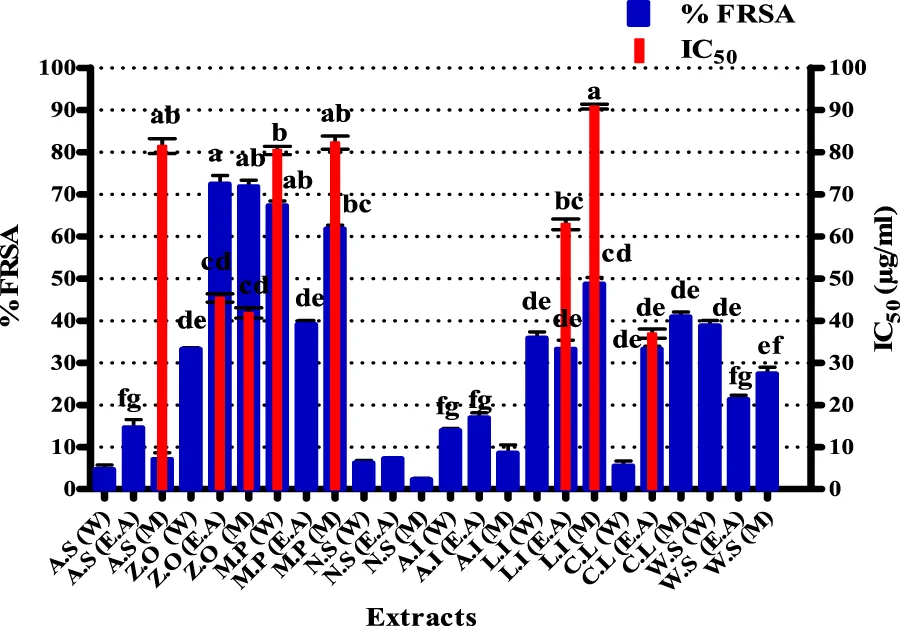
Free radical scavenging activity of test extracts. Values (mean ± SD) are the average of the triplicate analysis of each plant extract (*n* value of 1 × 3). The columns with different superscript (a-g) letters show significantly (*p* < 0.05) different means. FRSA, free radical scavenging activity; A. S, *Allium sativum*; Z. O, *Zingiber officinale*; M. P, *Mentha piperita*; N. S, *Nigella sativa*; L. I, *Lawsonia inermis;* C. L, *Curcuma longa*; W. S, *Withania somnifera*; EA, ethyl acetate extract; DW, distilled water extract; M, methanolic extract.

The results of the DPPH assay are presented in Figure 1. Graph Pad Prism 5 software was used to calculate the IC_50_ of samples. The maximum percent free radical scavenging activity was expressed by an M extract of *L. inermis* with an IC_50_ of 90.79 ± 0.58 μg/ml. Similarly, M and DW extracts of *M. piperita* expressed IC_50_ values of 82.23 ± 1.58 and 80.4 ± 0.9 μg/ml, respectively. The IC_50_ value of M extract of *A. sativum* was 81.43 ± 1.75 μg/ml. In the current study, the antioxidant profiling of the selected plants was achieved by employing a battery of assays, i.e., DPPH, total reducing power, and total antioxidant capacity assays. The procedures extrapolate antioxidant propensity by addressing various antioxidant mechanisms. The DPPH assay is based on the principle that when DPPH accepts a hydrogen (H) atom from the scavenger molecule, i.e., the antioxidant, the purple color changes to yellow, indicating the presence of 2, 2-diphenyl-1-picrylhydrazyl molecule (Mishra et al., 2012). Free radical scavenging activity in this study can be attributed to the presence of polyphenols such as catechin (Xu et al., 2011), gallic acid (Kim, 2007), syringic acid (Cikman et al., 2015), emodin (Vargas et al., 2004), luteolin (Roy et al., 2015), and quercetin (Ozgen et al., 2016) quantified in various extracts by HPLC analysis. In previously reported studies, a positive correlation has been observed between TPC and the DPPH assay, indicating the possible role of quantified polyphenols as free radical scavengers in the DPPH assay (Fernandes de Oliveira et al., 2012). Amplified amounts of reactive oxygen species (ROS) are a major cause of oxidative stress, which is an important factor in onychomycosis pathogenesis and may cause permanent tissue and organ injury (Zhang et al., 2021; Metin et al., 2021). Therefore, quenching of these ROS would be an important milestone to combat onychomycosis.

#### 4.1.2 Total antioxidant capacity

**FIGURE 2:**
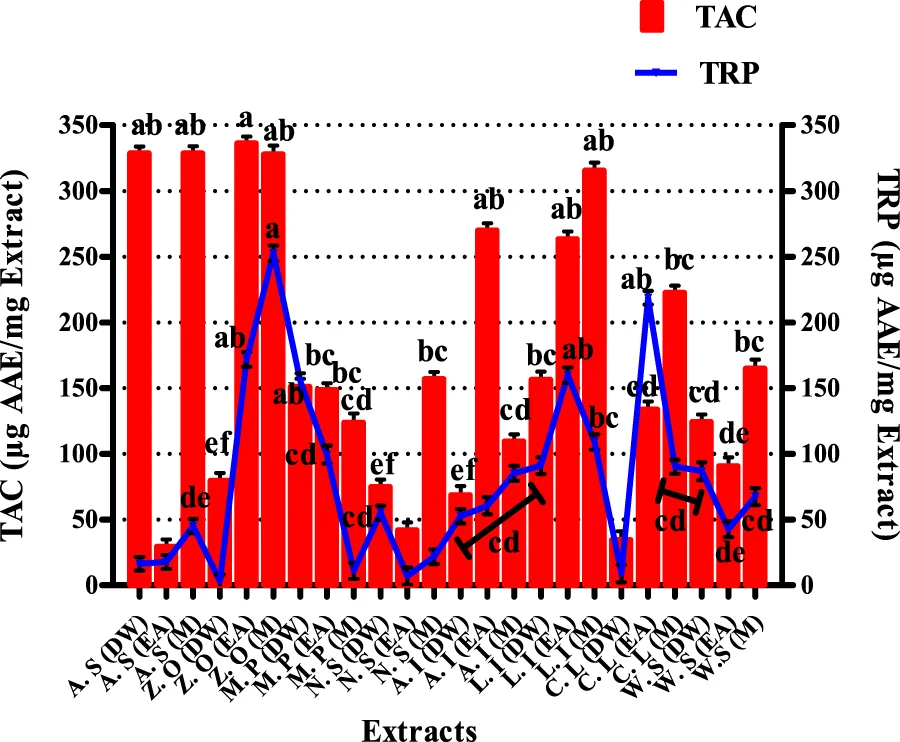
Graphical display of total antioxidant capacity and total reducing power of selected extracts. Values (mean ± SD) are the average of the triplicate analysis of each plant extract (*n* value of 1 × 3). The columns with different superscript (a–f) letters show significantly (*p* < 0.05) different means. A. S, *Allium sativum*; Z. O, *Zingiber officinale*; M. P, *Mentha piperita*; N. S, *Nigella sativa*; L. I, *Lawsonia inermis*; C. L, *Curcuma longa*; W. S, *Withania somnifera*; EA, ethyl acetate extract; DW, distilled water extract; M, methanolic extract.

The results of the TAC assay are expressed in microgram equivalent of ascorbic acid per milligram of extract presented in Figure 2. The most significant antioxidant potential noted was of DW and M extracts of *A. sativum,* which were 328.6 ± 5.08 and 328.64 ± 5.45 μg AAE/mgE, respectively. Similarly, EA and M extracts of *Z. officinale* exhibited maximum antioxidant capacity of 336.27 ± 5.19 and 327.76 ± 6.67 μg AAE/mgE, respectively. Likewise, EA and M extracts of *A. indica* showed total antioxidant capacity of 269.95 ± 5.55 and 109.45 ± 5.40 μg AAE/mg E, respectively. For *L. inermis,* the M extract exhibited the highest TAC of 315.43 ± 6.23 μg AAE/mg E. The total antioxidant capacity of the tested extracts is based on the sample reducing Phosphate-Mo (VI) to Phosphate-Mo (V) and forming a bluish-green colored phosphate/Mo (V) complex with acidic pH (Phatak and Hendre, 2014). As explained previously, the prominent antioxidant potential of the extracts makes them promising antioxidants against various infections, including onychomycosis, owing to their vital role in reducing oxidative stress, which is the foundation of many diseases due to its detrimental effects on the immune functions of biological systems (Apak et al., 2016).

#### 4.1.3 Total reducing power

The results of the total reducing power assay are graphically displayed in Figure 2 of extracts was expressed as ascorbic acid equivalent per milligram extract. The TRP results indicate that the EA extract of *C. longa* has shown a very proficient reducing power of 218.78 ± 5.23 μg AAE/mgE. EA and M extracts of *L. inermis* exhibited significant reducing power, i.e., 159.92 ± 5.89 and 109.13 ± 5.80 μg AAE/mgE respectively. DW extract of *M. piperita* expressed a substantial value of 155.50 ± 5.87 μg AAE/mgE. Likewise, EA and M extracts of *Z. officinale* also exhibited a significant reducing power of 252.73 ± 5.67 and 171.89 ± 5.88 μg AAE/mgE, respectively. The TRP assay is based on the ability of phenolic antioxidant test samples to reduce the ferric 2, 4, 6-tripyridyl-s-triazine complex [Fe_3_+-(TPTZ)_2_]^3+^ to the bright blue ferrous complex [Fe^2+^-(TPTZ)_2_]^2+^ in an acidic environment. The reducing properties are generally allied with the presence of reductones, which have been linked to the antioxidant action through breakage of the free radical chain by donating a hydrogen atom. Therefore a direct correlation has been observed between the antioxidant capacity and reducing power of certain plant extracts (Abdel-Hameed, 2009; Sathisha et al., 2011; Benslama and Harrar, 2016). The significant total reducing power of the tested extracts in the present study supports the aforementioned notion and denotes these extracts as useful candidates against onychomycosis by mitigating oxidative stress.

### 4.2 Phytochemical analysis

#### 4.2.1 Total phenolic content

**FIGURE 3:**
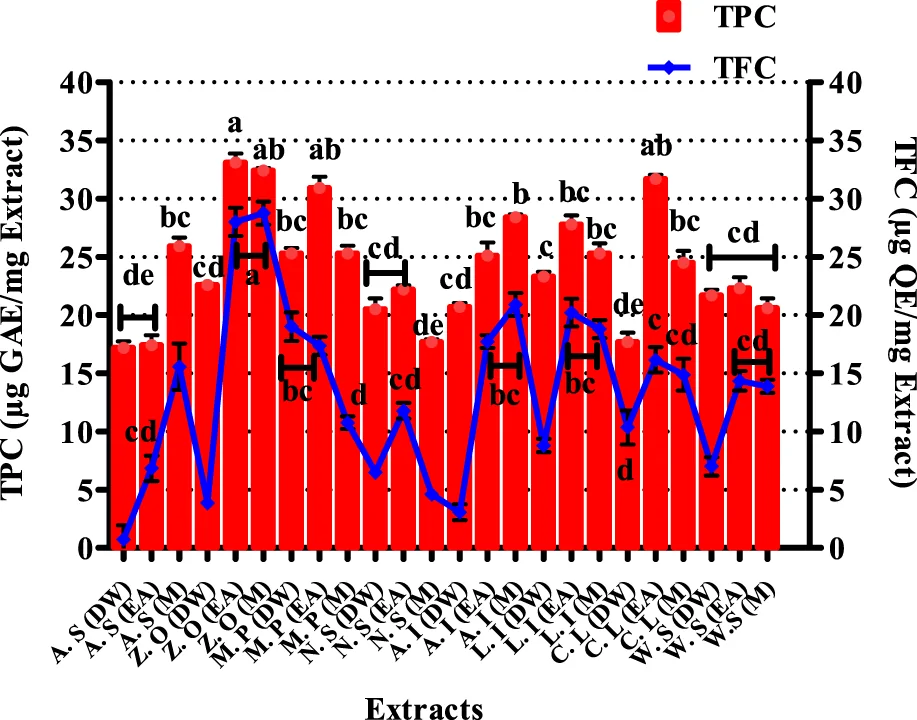
Graphical presentation of total phenolic (TPC) and total flavonoid content (TFC). Values (mean ± SD) are average of triplicate analysis of each plant extract (*n* value of 1 × 3). The columns with different superscript (a–e) letters show significantly (*p* < 0.05) different means. A. S, *Allium sativum*; Z. O, *Zingiber officinale*; M. P, *Mentha piperita*; N. S, *Nigella sativa*; L. I, *Lawsonia inermis;* C. L, *Curcuma longa*; W. S, *Withania somnifera*; EA, ethyl acetate extract; DW, distilled water extract; M, methanolic extract.

Total phenolic content estimation of selected plant extracts is shown in Figure 3. The highest phenolic content was found in *Z. officinale* EA and M extracts, i.e., 33.1 ± 0.76 and 32.4 ± 1.23 μg GAE/mgE, respectively. Quantification of TPC in *M. piperita* showed the EA extract to possess significant quantities of phenols (30.9 ± 0.98 μg GAE/mgE), while 31.7 ± 0.36 μg GAE/mgE was recorded in the EA extract of *C. longa*.

Immunosuppression increased oxidative stress in affected nail tissue (Zang et al., 2021), inflammation due to tissue injury (BARAN et al., 2021), and fungal infection (de Lima Grynszpan et al., 2021) are the contributing factors toward onychomycosis. Previously, various polyphenols have shown immunomodulatory (Yahfoufi et al., 2018), antifungal (Redondo-Blanco et al., 2020), antioxidant (Abbas et al., 2017), and anti-inflammatory properties (Cory et al., 2018). Therefore, in the current study, the total phenolic content of the tested extracts was quantified to assess their possible role in treating the said disease. Notable phenolic content as observed in these plants also emphasizes their role as capable contenders toward the treatment of onychomycosis owing to their aforementioned characteristics.

#### 4.2.2 Total flavonoid content

The TFC for various extracts of the selected plants is presented in Figure 3. The most significant quantity of flavonoids was measured in EA and M extracts of *Z. officinale,* i.e., 28.01 ± 1.22 μg and 28.75 ± 0.99 μg QE/mgE, respectively. The EA extract of *M. piperita* also showed noteworthy flavonoid content, i.e., 17.35 ± 0.77 μg QE/mgE. Similarly, the EA extract of *L. inermis* expressed a TFC of 20.21 ± 1.18 μg QE/mgE. Flavonoids protect plants from stress by scavenging reactive oxygen species (ROS) formed by the photosynthetic electron transport system (Agati et al., 2012). Flavonoids have also shown antioxidant (Agati et al., 2020), antifungal (Orhan et al., 2010), immunostimulatory (Talmale et al., 2014; Stroe and Oancea, 2020), anti-inflammatory (Serafini et al., 2010), and tissue healing attributes. They are also known to be effective inhibitors of various pro-inflammatory enzymes, such as cyclooxygenase (COX), lipoxygenase (LOX), xanthine oxidase (XO), and phosphoinositide 3-kinase (Hassan et al., 2022b). Therefore, the quantification of total flavonoids in the present study was performed to deduce their protective role in onychomycosis through their attributes. The results of the flavonoid content determination in the tested extracts suggest that they would be valuable antioxidants and would come forward as successful entrants against onychomycosis by strengthening the immunity through extenuation of oxidative stress.

#### 4.2.3 RP-HPLC analysis

**FIGURE 4:**
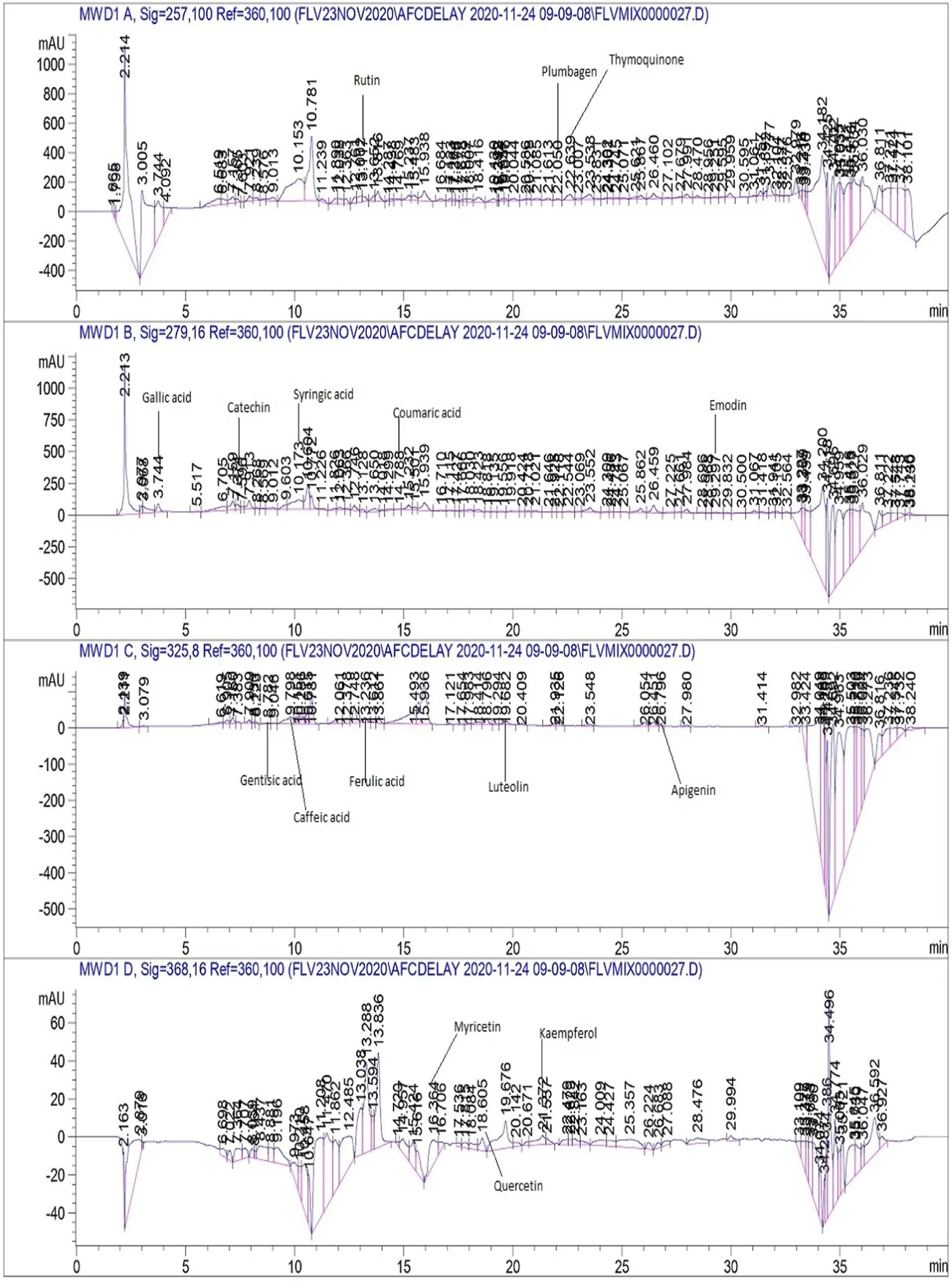
HPLC chromatogram of *L. inermis* M extract.

**FIGURE 5:**
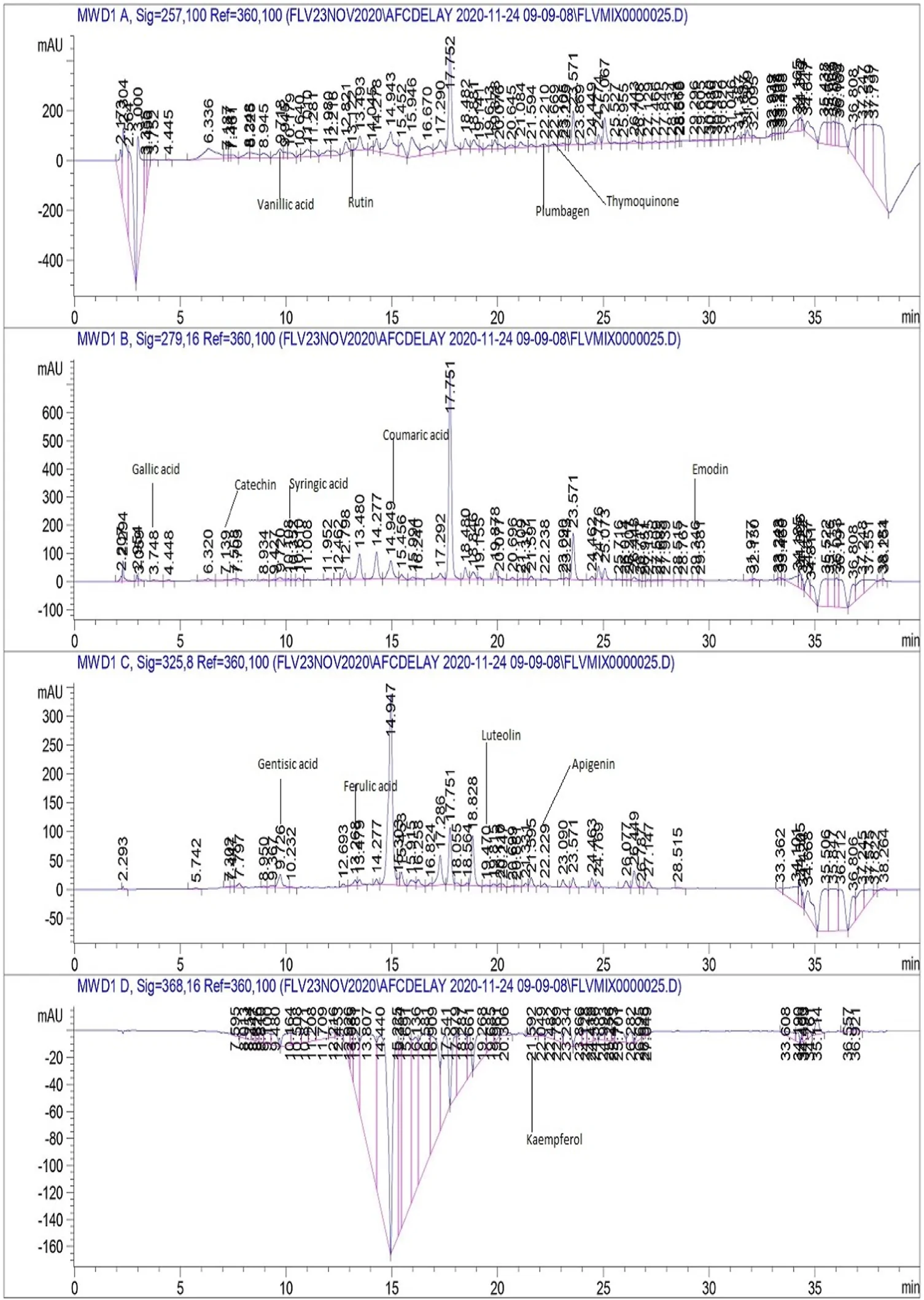
HPLC chromatogram of *M. piperita* M extract.

**FIGURE 6:**
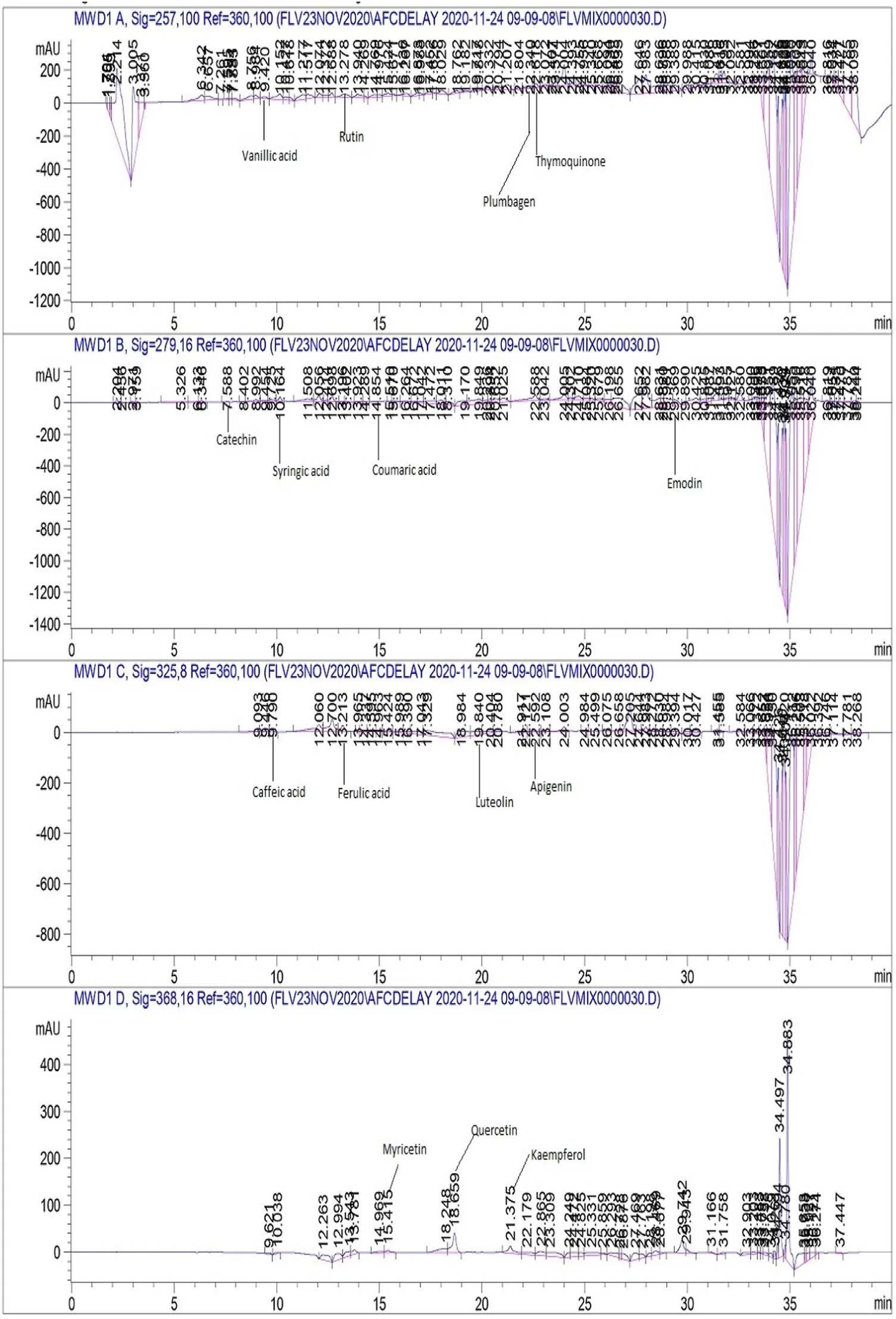
HPLC chromatogram of *A. indica* EA extract.

As maximum polyphenols were quantified in the EA and M extracts , they were proceeded with for the quantification of 17 therapeutically significant polyphenols with established antifungal activity, namely, vanillic acid, rutin, plumbagin, thymoquinone, gallic acid, catechin, syringic acid, coumaric acid, emodin, gentisic acid, caffeic acid, ferulic acid, luteolin, apigenin, myricetin, quercetin, and kaempferol were quantified (Table 3). Significant quantities of catechin and gallic acid were detected in the EA and M extracts of *L. inermis,* i.e., 93.04 ± 0.43 and 50.17 ± 0.22 μg/mg, respectively, and that of syringic acid was 55.63 ± 0.35 μg/mg in the M extract. Emodin was substantially quantified in the EA and M extracts of *L. inermis and C. longa* (246.32 ± 0.44 and 79.32 ± 0.33 μg/mg). In *A. indica,* it was 74.09 ± 0.24 μg/mg of the EA extract. Luteolin was 78.43 ± 0.18 μg/mg of the EA extract in *A. indica.* Quercetin was found to be 97.45 ± 0.22 μg/mg of the EA extract. RP-HPLC was performed to quantify the polyphenols in the selected plant extracts (EA and M) with significant antifungal activity against the causative agents of onychomycosis. Previous scientific investigation on the possible antifungal action of catechin shows that it disturbs the synthesis of the cell wall, damages the plasma membrane, and causes the lysis of hyphae and spores of the fungus (Yang and Jiang, 2015). Gallic acid shows antifungal action by reducing the activity of enzymes such as sterol 14α-demethylase P450 (CYP51) and squalene epoxidase in the fungal membrane (Li et al., 2017), while syringic acid, vanillic acid, and emodin show antifungal action by inhibiting the peroxidase and the fungal α-amylase enzyme (Silva et al., 2020; Janeczko, 2018). Luteolin shows antifungal action by inducing apoptosis (Seleem et al., 2017). Quercetin shows antifungal action by affecting the ergosterol of the fungal cell wall (Bitencourt et al., 2013), whereas kaempferol and rutin cause cell wall lysis and interference of microbial protein synthesis, respectively (Bae et al., 1997; Reddy et al., 2016). Caffeic acid shows the antifungal action by fungal 1,3-β-d-glucan synthase inhibition (Ma and Ma, 2015). Therefore, substantial quantification of these polyphenols in the extracts indicates their possible antifungal action and ultimately their effectiveness in the treatment plan against onychomycosis. Figures 4, 5 display the chromatograms of the M extracts of *L. inermis* and *M. piperita,* and Figure 6 shows the EA extract of *A. indica*.

**TABLE 3:** HPLC based quantification of polyphenols in selected plant extracts.

Concentration (µg/mg of extract)																	
Phenols	RT (min)	*Allium sativum*		*Zingiber officinale*		*Withania somnifera*		*Curcuma longa*		*Mentha piperita*		*Lawsonia inermis*		*Nigella sativa*		*Azadirachta indica*	
		EA	M	EA	M	EA	M	EA	M	EA	M	EA	M	EA	M	EA	M
Vanillic acid	9.54	6.28 ± 0.01	1.94 ± 0.02	7.85 ± 0.11	3.20 ± 0.01	8.31 ± 0.01	1.77 ± 0.01	2.51 ± 0.10	1.87 ± 0.01	3.20 ± 0.01	5.39 ± 0.01	2.41 ± 0.01	Nd	2.99 ± 0.12	4.84 ± 0.01	1.10 ± 0.01	4.55 ± 0.11
Rutin	13.63	14.19 ± 0.11	20.06 ± 0.05	5.66 ± 0.03	6.99 ± 0.01	Nd	16.98 ± 0.03	8.03 ± 0.11	35.71 ± 0.23	61.6 ± 0.23	0.89 ± 0.01	Nd	29.54 ± 0.11	19.01 ± 0.22	4.12 ± 0.01	23.74 ± 0.22	Nd
Plumbagin	22.39	0.93 ± 0.02	1.31 ± 0.03	4.18 ± 0.03	5.10 ± 0.01	Nd	3.02 ± 0.02	13.27 ± 0.12	1.38 ± 0.01	3.00 ± 0.01	3.24 ± 0.02	3.98 ± 0.01	2.02 ± 0.01	2.18 ± 0.10	1.77 ± 0.11	5.02 ± 0.11	1.81 ± 0.01
Thymoquinone	22.65	0.66 ± 0.09	0.45 ± 0.01	1.53 ± 0.02	1.88 ± 0.01	11.31 ± 0.01	7.89 ± 0.02	Nd	0.77 ± 0.01	1.04 ± 0.01	1.24 ± 0.02	1.37 ± 0.01	6.54 ± 0.21	4.01 ± 0.11	1.58 ± 0.02	2.69 ± 0.11	1.43 ± 0.01
Gallic acid	3.72	Nd	Nd	Nd	2.64 ± 0.02	Nd	1.30 ± 0.01	Nd	Nd	Nd	2.42 ± 0.02	22.24 ± 0.21	50.17 ± 0.22	Nd	Nd	Nd	Nd
Catechin	7.33	Nd	Nd	Nd	Nd	1.12 ± 0.01	Nd	Nd	1.34 ± 0.01	Nd	5.79 ± 0.03	93.04 ± 0.43	29.58 ± 0.23	Nd	Nd	1.54 ± 0.11	2.01 ± 0.02
Syringic acid	10.00	Nd	0.74 ± 0.01	0.33 ± 0.01	1.38 ± 0.03	3.21 ± 0.01	0.43 ± 0.01	29.32 ± 0.24	2.10 ± 0.01	3.69 ± 0.01	1.03 ± 0.02	0.50 ± 0.01	55.63 ± 0.35	Nd	1.60 ± 0.11	5.56 ± 0.11	1.85 ± 0.04
Coumaric acid	14.76	Nd	Nd	Nd	Nd	0.21 ± 0.01	Nd	0.36 ± 0.01	Nd	0.72 ± 0.01	2.85 ± 0.03	Nd	0.19 ± 0.10	Nd	Nd	0.08 ± 0.01	Nd
Emodin	29.05	28.71 ± 0.6	21.17 ± 0.06	171.92 ± 0.22	37.27 ± 0.23	Nd	44.73 ± 0.17	36.58 ± 0.34	79.32 ± 0.33	5.50 ± 0.02	3.26 ± 0.03	246.32 ± 0.44	15.58 ± 0.04	2.12 ± 0.01	Nd	74.09 ± 0.24	22.20 ± 0.33
Gentisic acid	8.08	Nd	Nd	Nd	Nd	Nd	Nd	Nd	Nd	Nd	33.35 ± 0.35	5.38 ± 0.21	2.03 ± 0.11	Nd	Nd	Nd	Nd
Caffeic acid	9.59	0.40 ± 0.07	Nd	Nd	Nd	1.15 ± 0.11	Nd	Nd	Nd	Nd	Nd	2.53 ± 0.02	12.98 ± 0.22	Nd	1.21 ± 0.22	2.79 ± 0.10	1.04 ± 0.01
Ferulic acid	13.08	0.16 ± 0.01	0.37 ± 0.02	0.36 ± 0.02	0.40 ± 0.04	0.74 ± 0.04	0.25 ± 0.02	4.53 ± 0.27	1.59 ± 0.11	0.50 ± 0.05	0.53 ± 0.02	0.77 ± 0.01	0.96 ± 0.02	Nd	Nd	0.20 ± 0.01	0.39 ± 0.01
Luteolin	19.58	Nd	Nd	Nd	Nd	3.20 ± 0.14	5.88 ± 0.11	Nd	Nd	12.34 ± 0.02	3.83 ± 0.02	24.28 ± 0.12	28.25 ± 0.11	Nd	Nd	78.43 ± 0.18	Nd
Apigenin	22.02	Nd	Nd	Nd	Nd	2.71 ± 0.13	0.75 ± 0.12	7.15 ± 0.16	Nd	2.98 ± 0.03	1.15 ± 0.03	3.39 ± 0.02	2.78 ± 0.01	Nd	Nd	0.94 ± 0.01	Nd
Myricetin	15.5	Nd	Nd	Nd	Nd	1.93 ± 0.11	8.23 ± 0.14	1.85 ± 0.01	Nd	7.64 ± 0.01	29.44 ± 0.13	0.83 ± 0.01	1.29 ± 0.02	Nd	Nd	0.58 ± 0.02	0.42 ± 0.05
Quercetin	18.51	Nd	8.31 ± 0.02	Nd	Nd	38.69 ± 0.09	Nd	10.12 ± 0.11	Nd	24.27 ± 0.11	57.45 ± 0.16	Nd	14.93 ± 0.02	Nd	Nd	97.45 ± 0.22	14.39 ± 0.02
Kaempferol	21.27	Nd	Nd	3.04 ± 0.07	1.08 ± 0.02	17.75 ± 0.12	2.81 ± 0.11	5.11 ± 0.02	Nd	13.38 ± 0.01	8.20 ± 0.01	2.32 ± 0.01	7.01 ± 0.03	Nd	Nd	16.99 ± 0.04	5.52 ± 0.02

Nd, not detected; RT, retention time; EA, ethanolic extract; M, methanolic extract.

### 4.3 Preliminary resistance profiling

The susceptibility of the selected clinical isolates of dermatophytes (*T. rubrum*, *T. tonsurans,* and *A. alternata*) and non-dermatophytes (*A. niger*, *A. flavus*, *F. dimerum*, *R. arrhizus*, and *A. terreus*) against contemporary antifungals (terbinafine and amphotericin-B) was assessed by the microbroth dilution method (Table 4). Results showed that the growth of dermatophyte clinical isolates was inhibited by terbinafine, and amphotericin-B had no effect on them whatsoever and that all non-dermatophyte clinical isolates were resistant to terbinafine and inhibited by amphotericin-B. Hence, terbinafine was used in combination with the tested extracts for synergistic studies against dermatophytes, and amphotericin-B was used for combination studies against non-dermatophyte clinical isolates.

**TABLE 4:** Resistance profiling of selected antifungals against dermatophyte and non-dermatophyte clinical isolates.

Selected antifungals	Antifungal Activity							
	T. R	T. T	Al. A	A. T	Rh	A. F	A. N	F. D
Terbinafine	S	S	S	R	R	R	R	R
Amphotericin-B	R	R	R	S	S	S	S	S

T. R, *Trichophyton rubrum*; T. T, *Trichophyton tonsurans*; Al. A, *Alternaria alternata*; Rh, *Rhizopus arrhizus*; A. F, *Aspergillus flavus*; A. N, *Aspergillus niger*; F. D, *Fusarium dimerum*; S, sensitive; *R, resistant*.

### 4.4 Antifungal susceptibility testing

The results of antifungal susceptibility testing (AST) against dermatophytes and non-dermatophytes and against *C. albicans* are presented in Table 5. The DW extract of *A. sativum* showed significant activity against *R. arrhizus* with an MIC of 31.25 ± 0.31 μg/ml. EA, M, and DW extracts of *Z. officinale, N. sativa,* and *C. longa,* while EA and DW extracts of *M. piperita* exhibited promising activity against different strains with an MIC of 31.25 μg/ml. Extracts of *L. inermis*, *C. longa,* and *N.* sativa are more active against dermatophytes. These extracts showed no significant activity against *C. albicans.* As reported earlier, well-known plant secondary metabolites exhibiting antifungal activity include flavonoids, phenols, and phenolic glycosides. Therefore, in our present study, the antifungal activity might be attributed to the presence of antioxidant moieties as shown by the results of antioxidant assays *via* amelioration of oxidative stress along with phenolic compounds quantified through HPLC such as catechin (Yang and Jiang, 2015), gallic acid (Li et al., 2017), syringic acid, emodin, vanillic acid (Silva et al., 2020; Janeczko, 2018), quercetin (Bitencourt et al., 2013), luteolin (Seleem et al., 2017), kaempferol, and rutin (Bae et al., 1997; Reddy et al., 2016).

**TABLE 5:** Antifungal potential of tested extracts against dermatophytes and non-dermatophytes.

Extracts	Antifungal assay							
	Minimum inhibitory concentration (µg/ml) (mean ± SD)*							
	T. R	T. T	Al. A	A. T	Rh	A. F	A. N	F. D
*Allium sativum*								
EA	62.5 ± 0.11	62.5 ± 0.21	125 ± 0.22	125 ± 0.22	125 ± 0.11	--	500 ± 0.22	125 ± 0.26
M	62.5 ± 0.13	62.5 ± 0.22	31.25 ± 0.23	--	125 ± 0.21	--	500 ± 0.25	125 ± 0.31
DW	62.5 ± 0.11	125 ± 0.21	62.5 ± 0.19	125 ± 0.22	31.25 ± 0.31	125 ± 0.15	500 ± 0.22	31.25 ± 0.32
*Zingiber officinale*								
EA	62.5 ± 0.23	125 ± 0.25	125 ± 0.26	31.25 ± 0.34	62.5 ± 0.26	250 ± 0.22	500 ± 0.28	62.5 ± 0.14
M	31.25 ± 0.25	250 ± 0.22	125 ± 0.25	62.5 ± 0.27	62.5 ± 0.21	--	500 ± 0.34	62.5 ± 0.11
DW	--	125 ± 0.21	500 ± 0.34	125 ± 0.26	31.25 ± 0.26	31.25 ± 0.24	500 ± 0.31	31.25 ± 0.31
*Mentha piperita*								
EA	125 ± 0.33	62.5 ± 0.22	250 ± 0.29	--	31.25 ± 0.32	--	500 ± 0.26	125 ± 0.17
M	125 ± 0.23	62.5 ± 0.34	62.5 ± 0.26	125 ± 0.36	125 ± 0.18	500 ± 0.14	--	250 ± 0.21
DW	125 ± 0.22	31.25 ± 0.45	250 ± 0.31	--	125 ± 0.36	250 ± 0.23	500 ± 0.27	125 ± 0.26
*Nigella sativa*								
EA	62.5 ± 0.25	31.26 ± 0.34	62.5 ± 0.23	125 ± 0.22	31.25 ± 0.17	--	125 ± 0.29	31.25 ± 0.21
M	31.25 ± 0.25	62.5 ± 0.22	--	250 ± 0.27	31.25 ± 0.31	500 ± 0.34	--	31.25 ± 0.23
DW	62.5 ± 0.19	125 ± 0.51	62.5 ± 0.29	--	31.25 ± 0.24	--	--	31.25 ± 0.34
*Azadirachta indica*								
EA	62.5 ± 0.16	250 ± 0.32	62.5 ± 0.44	125 ± 0.34	62.5 ± 0.21	62.5 ± 0.31	500 ± 0.27	62.5 ± 0.32
M	62.5 ± 0.23	125 ± 0.29	125 ± 0.36	125 ± 0.34	62.5 ± 0.25	500 ± 0.33	--	62.5 ± 0.32
DW	62.5 ± 0.31	250 ± 0.16	31.25 ± 0.21	250 ± 0.17	125 ± 0.28	62.5 ± 0.13	62.5 ± 0.35	125 ± 0.34
*Lawsonia inermis*								
EA	62.5 ± 0.35	62.5 ± 0.23	62.5 ± 0.34	125 ± 0.32	125 ± 0.25	125 ± 0.35	125 ± 0.29	62.5 ± 0.15
M	125 ± 0.16	31.25 ± 0.15	125 ± 0.34	250 ± 0.21	125 ± 0.26	250 ± 0.33	250 ± 0.31	125 ± 0.23
DW	62.6 ± 0.11	125 ± 0.29	125 ± 0.25	125 ± 0.24	62.5 ± 0.31	--	--	62.5 ± 0.36
*Curcuma longa*								
EA	62.6 ± 0.24	125 ± 0.15	250 ± 0.31	31.25 ± 0.22	125 ± 0.41	250 ± 0.35	125 ± 0.36	62.5 ± 0.36
M	31.25 ± 0.27	31.25 ± 0.24	125 ± 0.17	--	125 ± 0.26	125 ± 0.26	250 ± 0.31	125 ± 0.26
DW	31.25 ± 0.13	31.25 ± 0.18	250 ± 0.21	62.5 ± 0.32	--	62.5 ± 0.27	250 ± 0.28	62.5 ± 28
*Withania somnifera*								
EA	125 ± 0.25	125 ± 0.28	62.5 ± 0.35	125 ± 0.33	62.5 ± 0.22	250 ± 0.26	250 ± 0.31	125 ± 0.31
M	125 ± 0.33	125 ± 0.31	125 ± 0.29	125 ± 0.28	62.5 ± 0.34	500 ± 0.22	250 ± 0.23	125 ± 0.33
DW	125 ± 0.28	250 ± 0.33	125 ± 0.31	125 ± 0.32	62.5 ± 0.41	250 ± 0.27	500 ± 0.28	125 ± 0.36
Controls								
Terb	0.005 ± 0.11	0.005 ± 0.13	0.005 ± 0.11	--	--	--	--	--
Amp B	--	--	--	3.2 ± 0.12	3.2 ± 0.01	3.2 ± 0.12	3.2 ± 0.11	3.2 ± 0.09
DMSO	--	--	--	--	--	--	--	--
	Candida albicans**							
	*Diameter of zone of inhibition* (*mm*) (*mean ±SD*)*							
	*A. sativum*	*Z. officinale*	*M. piperita*	*N. sativa*	*A. indica*	*L. inermis*	*C. longa*	*W. somnifera*
EA	**--**	**--**	--	--	--	3 ± 0.9	**--**	**--**
M	6 ± 0.2	**--**	--	--	--	--	5 ± 0.4	**--**
DW	**--**	**--**	--	--	--	--	**--**	**--**
Controls								
Amp-B	17 ± 0.54	DMSO	--					

Values (mean ± SD) are average of triplicate analysis of each plant extract (n value of 1 × 3). The sample concentration was 500, 250, 125, 62.5, and 31.25 μg/ml in each corresponding well of the 96-well plate. EA, ethyl acetate extract, M, methanolic extract; DW, distilled water extract; --, No activity; T.R, *Trichophyton rubrum*; T. T, *Trihcophyton tonsurans*; Al. A, *Alternaria alternata*; Rh, *Rhizopus arrhizus*; A. F, *Aspergillus flavus;* A. N, *Aspergillus niger*; F. D, *Fusarium dimerum*; Terb, terbinafine; Amp-B, amphotericin B; **, disc diffusion method was applied for screening against *Candida albicans*.

### 4.5 Evaluation of synergistic interactions between extracts and standard antifungals

The standard checkerboard method was employed for evaluation of synergistic interactions between the tested extracts and the standard antifungals. The results showed a significant synergy, as indicated by the lowering of the MICs of conventional antifungals by 4- and 8-fold (Table 6). For *T. rubrum*, the M and DW extracts of *Z. officinale* have shown 8-fold reductions in the MIC values when treated alone or in combination with terbinafine. For *T. tonsurans*, DW extract of *M. piperita*, *L. inermis,* and *N. sativa,* whereas M extract of *N. sativa*, *A. sativum, L. inermis,* and *Z. officinale* have shown 8-fold reductions in MICs of both extracts and terbinafine. Similarly, the EA extract of *C. longa* against *A. niger* has shown 8-fold reductions in the MICs of both extracts and AMB. Against *F. dimerum*, the M extract of *L. inermis* exhibited 8-fold reductions in MICs for both the extract and AMB. The checkerboard method permits the assessment of the concentration of antimicrobials able to remove microorganisms at a stated incubation time. The combination of antimicrobials with polyphenols can prove to be very advantageous (Zacchino et al., 2017; Song and Wu., 2022). Earlier studies have reported that flavonoids have considerable antibacterial and antifungal potentials and when used in combination with antimicrobials can produce synergistic performance and thus boost the whole effect counter to infectious diseases (Al Aboody and Mickymaray, 2020). Because plant metabolites are not part of standard therapy, they can be contemplated as monotherapy or in combination therapy against microbial diseases (Amin et al., 2015). Thus, the synergistic interactions in this study might be due to the antifungal activities of extracts containing antifungal phenolics such as vanillic acid (Silva et al., 2020), kaempferol, rutin (Bae et al., 1997; Reddy et al., 2016), quercetin (Bitencourt et al., 2013), luteolin, gallic acid (Li et al., 2017) and syringic acid (Silva et al., 2020) quantified in tested extracts with documented antifungal properties as previously stated (Section 4.3) and the antifungal action of reference drugs utilized (Ryder, 1992) (Mesa-Arango et al., 2012), which makes these extracts even more suitable to be selected as alternative anti-onychomycotic agents (Zhang et al., 2022). To the best of our knowledge, determination of synergistic interactions between these extracts and commercial antifungals has been carried out for the first time in this study.

**TABLE 6:** Representation of synergistic interactions between extracts and antifungal drugs.

Tested extracts	MIC alone µg/mL	MIC combination µg/mL	FICI value	Fold reductions	Interpretation
*Trichophyton rubrum*					
A. S (M)	31.25	7.8	0.365	4	Synergy
Terbinafine	0.005	0.000625		8	
A. S (DW)	31.25	3.9	0.374	8	Synergy
Terbinafine	0.005	0.00125		4	
Z. O (EA)	31.25	7.8	0.365	4	Synergy
Terbinafine	0.005	0.000625		8	
Z. O (M)	31.25	3.9	0.249	8	Synergy
Terbinafine	0.005	0.000625		8	
Z. O (DW)	31.25	3.9	0.249	8	Synergy
Terbinafine	0.005	0.000625		8	
M. P (DW)	31.25	3.9	0.624	8	Additive
Terbinafine	0.005	0.0025		2	
N. S (M)	31.25	15.6	0.9	2	Additive
Terbinafine	0.005	0.0025		2	
A. I (DW)	31.25	7.8	0.365	4	Synergy
Terbinafine	0.005	0.00625		8	
C. L (EA)	31.25	31.25	1.125	0	Indifferent
Terbinafine	0.005	0.00625		8	
C. L (M)	31.25	62.5	2.125	0	Indifferent
Terbinafine	0.005	0.000625		8	
*Trichophyton tonsurans*					
A. S (M)	31.25	3.9	0.249	8	Synergy
Terbinafine	0.005	0.000625		8	
A. S (DW)	31.25	7.8	0.365	4	Synergy
Terbinafine	0.005	0.000625		8	
Z. O (EA)	31.25	3.9	0.374	8	Synergy
Terbinafine	0.005	0.00125		4	
Z. O (M)	31.25	3.9	0.249	8	Synergy
Terbinafine	0.005	0.000625		8	
Z. O (DW)	31.25	15.6	0.525	2	Synergy
Terbinafine	0.005	0.000625		8	
M. P (DW)	31.25	3.9	0.249	8	Synergy
Terbinafine	0.005	0.000625		8	
N. S (EA)	31.25	7.8	0.365	4	Synergy
Terbinafine	0.005	0.000625		8	
N. S (M)	31.25	3.9	0.249	8	Synergy
Terbinafine	0.005	0.000625		8	
N. S (DW)	31.25	3.9	0.249	8	Synergy
Terbinafine	0.005	0.000625		8	
A. I (DW)	31.25	7.8	0.365	4	Synergy
Terbinafine	0.005	0.000625		8	
L. I (M)	31.25	3.9	0.249	8	Synergy
Terbinafine	0.005	0.000625		8	
L. I (DW)	31.25	3.9	0.249	8	Synergy
Terbinafine	0.005	0.000625		8	
C. L (EA)	31.25	15.6	0.65	2	Additive
Terbinafine	0.005	0.00125		2	
C. L (M)	31.25	3.9	0.374	8	Synergy
Terbinafine	0.005	0.00125		4	
C. L (DW)	31.25	7.8	0.365	4	Synergy
Terbinafine	0.005	0.000625		8	
*Alternaria alternata*					
A. S (M)	31.25	3.9	0.249	8	Synergy
Terbinafine	0.005	0.000625		8	
A. S (DW)	31.25	3.9	0.249	8	Synergy
Terbinafine	0.005	0.000625		8	
Z. O (EA)	31.25	3.9	0.249	8	Synergy
Terbinafine	0.005	0.000625		8	
Z. O (M)	31.25	3.9	0.249	8	Synergy
Terbinafine	0.005	0.000625		8	
Z. O (DW)	31.25	31.25	1.125	0	Indifferent
Terbinafine	0.005	0.000625		8	
M. P (DW)	31.25	7.8	0.365	4	Synergy
Terbinafine	0.005	0.000625		8	
N. S (EA)	31.25	7.8	0.49	4	Synergy
Terbinafine	0.005	0.00125		8	
N. S (M)	31.25	62.5	2.125	0	Indifferent
Terbinafine	0.005	0.000625		8	
N. S (DW)	31.25	15.6	0.525	2	Synergy
Terbinafine	0.005	0.000625		8	
A. I (DW)	31.25	15.6	0.525	2	Synergy
Terbinafine	0.005	0.000625		8	
L. I (M)	31.25	7.8	0.365	4	Synergy
Terbinafine	0.005	0.000625		8	
L. I (DW)	31.25	31.25	1.125	0	Indifferent
Terbinafine	0.005	0.000625		8	
C. L (EA)	31.25	31.25	1.25	0	Indifferent
Terbinafine	0.005	0.00125		2	
C. L (M)	31.25	31.25	1.125	0	Indifferent
Terbinafine	0.005	0.000625		8	
C. L (DW)	31.25	15.6	0.65	2	Additive
Terbinafine	0.005	0.00125		2	
*Aspergillus terreus*					
N. S (M)	31.25	62.5	2.15	0	Indifferent
Amp- B	3.2	1.6		2	
N. S (DW)	31.25	62.5	2.125	0	Indifferent
Amp- B	3.2	0.4		8	
L. I (M)	31.25	15.6	0.9	2	Additive
Amp- B	3.2	1.6		2	
L. I (DW)	31.25	31.25	1.5	0	Indifferent
Amp- B	3.2	1.6		2	
C. L (DW)	31.25	7.8	1.24	4	Indifferent
Amp- B	3.2	3.2		0	
*Rhizopus arrhizus*					
A. S (M)	31.25	7.8	0.49	4	Synergy
Amp- B	3.2	0.8		4	
A. S (DW)	31.25	7.8	0.365	4	Synergy
Amp- B	3.2	0.4		8	
Z. O (EA)	31.25	31.25	1.125	0	Indifferent
Amp- B	3.2	0.4		8	
Z. O (M)	31.25	15.6	0.52	2	Synergy
Amp- B	3.2	0.4		8	
Z. O (DW)	31.25	31.25	1.125	0	Indifferent
Amp- B	3.2	0.4		8	
M. P (DW)	31.25	15.6	0.52	2	Synergy
Amp- B	3.2	0.4		8	
N. S (EA)	31.25	3.9	0.245	8	Synergy
Amp- B	3.2	0.4		8	
N. S (M)	31.25	7.8	0.365	4	Synergy
Amp- B	3.2	0.4		8	
N. S (DW)	31.25	3.9	0.245	8	Synergy
Amp- B	3.2	0.4		8	
A. I (DW)	31.25	31.25	1.125	0	Indifferent
Amp- B	3.2	0.4		8	
L. I (M)	31.25	3.9	0.245	8	Synergy
Amp- B	3.2	0.4		8	
L. I (DW)	31.25	7.8	0.365	4	Synergy
Amp- B	3.2	0.4		8	
C. L (EA)	31.25	3.9	0.245	8	Synergy
Amp- B	3.2	0.4		8	
C. L (M)	31.25	3.9	0.374	8	Synergy
Amp- B	3.2	0.8		4	
C. L (DW)	31.25	31.25	1.5	0	Indifferent
Amp- B	3.2	1.6		2	
*Aspergillus flavus*					
A. S (M)	31.25	15.6	0.65	2	Additive
Amp- B	3.2	0.8		8	
A. S (DW)	31.25	15.6	0.9	2	Additive
Amp- B	3.2	1.6		2	
M. P (DW)	31.25	3.9	0.245	8	Synergy
Amp- B	3.2	0.4		8	
A. I (DW)	31.25	15.6	1.4	2	Indifferent
Amp- B	3.2	3.2		0	
L. I (M)	31.25	3.9	0.245	8	Synergy
Amp- B	3.2	0.4		8	
L. I (DW)	31.25	3.9	0.245	8	Synergy
Amp- B	3.2	0.4		8	
C. L (M)	31.25	15.6	0.65	2	Additive
Amp- B	3.2	0.8		4	
*Aspergillus niger*					
A. S (M)	31.25	7.8	0.374	4	Synergy
Amp- B	3.2	0.4		8	
A. S (DW)	31.25	31.24	1.125	0	Indifferent
Amp- B	3.2	0.4		8	
Z. O (EA)	31.25	15.6	0.52	2	Synergy
Amp- B	3.2	0.4		8	
Z. O (M)	31.25	15.6	0.52	2	Synergy
Amp- B	3.2	0.4		8	
Z. O (DW)	31.25	62.5	2.15	0	Indifferent
Amp- B	3.2	1.6		2	
M. P (DW)	31.25	31.25	1.125	0	Indifferent
Amp- B	3.2	0.4		8	
N. S (EA)	31.25	15.6	0.52	2	Synergy
Amp- B	3.2	0.4		8	
N. S (M)	31.25	31.25	1.125	0	Indifferent
Amp- B	3.2	0.4		8	
N. S (DW)	31.25	15.6	0.52	2	Synergy
Amp- B	3.2	0.4		8	
L. I (M)	31.25	62.5	2.25	0	Indifferent
Amp- B	3.2	0.8		4	
L. I (DW)	31.25	15.6	0.9	2	Additive
Amp- B	3.2	1.6		2	
C. L (EA)	31.25	3.9	0.245	8	Synergy
Amp- B	3.2	0.4		8	
C. L (M)	31.25	7.8	0.365	4	Synergy
Amp- B	3.2	0.4		8	
C. L (DW)	31.25	31.25	1.125	0	Indifferent
Amp- B	3.2	0.4		8	
*Fusarium dimerum*					
Z. O (M)	31.25	31.25	1.25	0	Indifferent
Amp- B	3.2	0.8		4	
M. P (DW)	31.25	15.6	0.65	2	Additive
Amp- B	3.2	0.8		4	
N. S (DW)	31.25	31.25	1.25	0	Indifferent
Amp- B	3.2	0.8		4	
L. I (M)	31.25	3.9	0.24	8	Synergy
Amp- B	3.2	0.4		8	
L. I (DW)	31.25	3.9	0.374	8	Synergy
Amp- B	3.2	0.8		4	
C. L (M)	31.25	15.6	0.9	2	Additive
Amp- B	3.2	1.6		2	

MIC, minimum inhibitory concentrations; FICI, fractional inhibitory concentration index; A. S, *Allium sativum*; Z. O, *Zingiber officinale*; M. P, *Mentha piperita*; N. S, *Nigella sativa*; A. I, *Azadirachta indica*; L. I, *Lawsonia inermis*; C. L, *Curcuma longa*; EA, ethyl acetate extract; M, methanolic extract; DW, distilled water extract; Amp-B, amphotericin-B.

### 4.6 Time-kill kinetics

**FIGURE 7:**
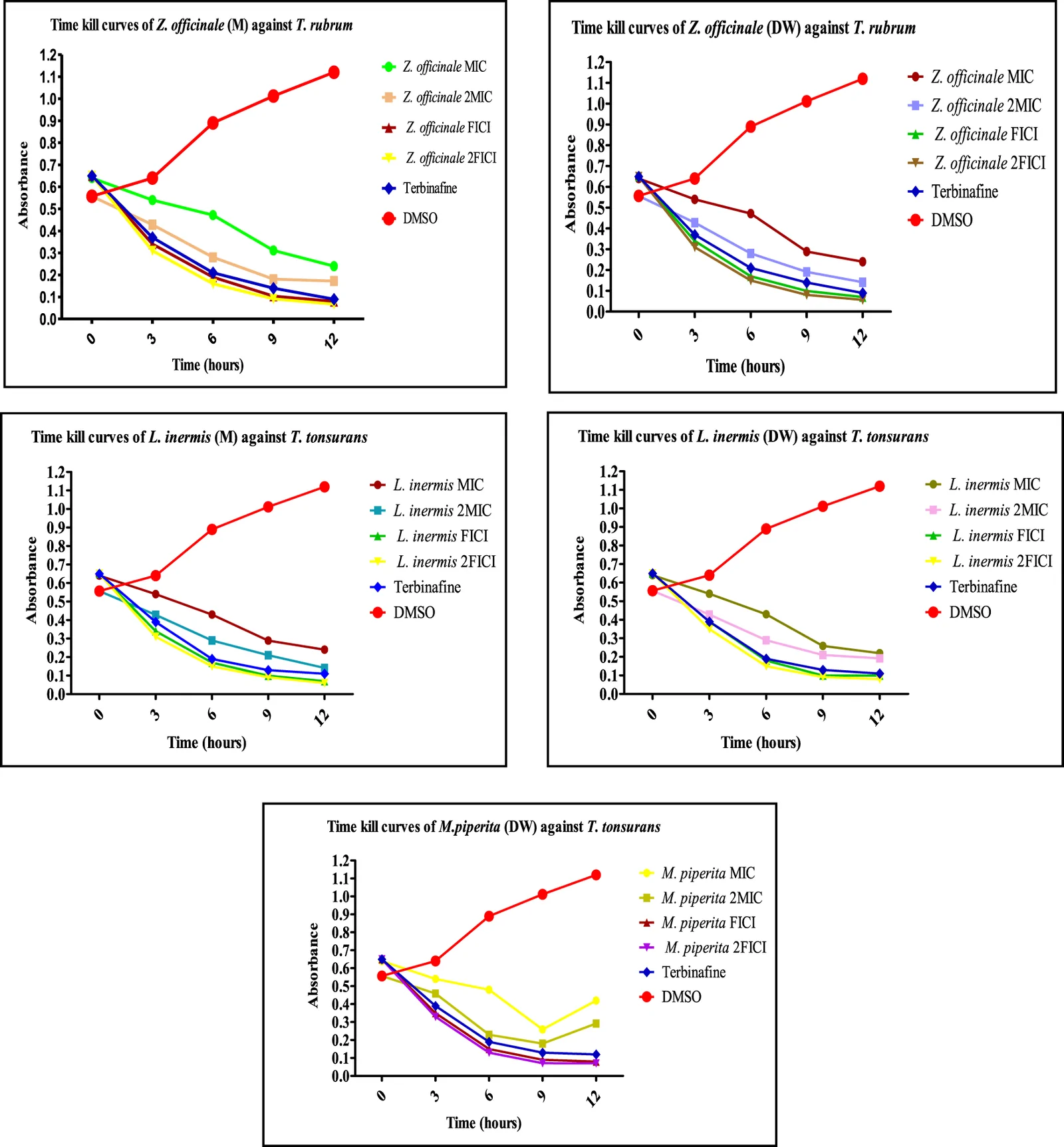
Time-kill curves of selected extracts against dermatophytes. M, methanolic extract; DW, distilled water extract.

**FIGURE 8:**
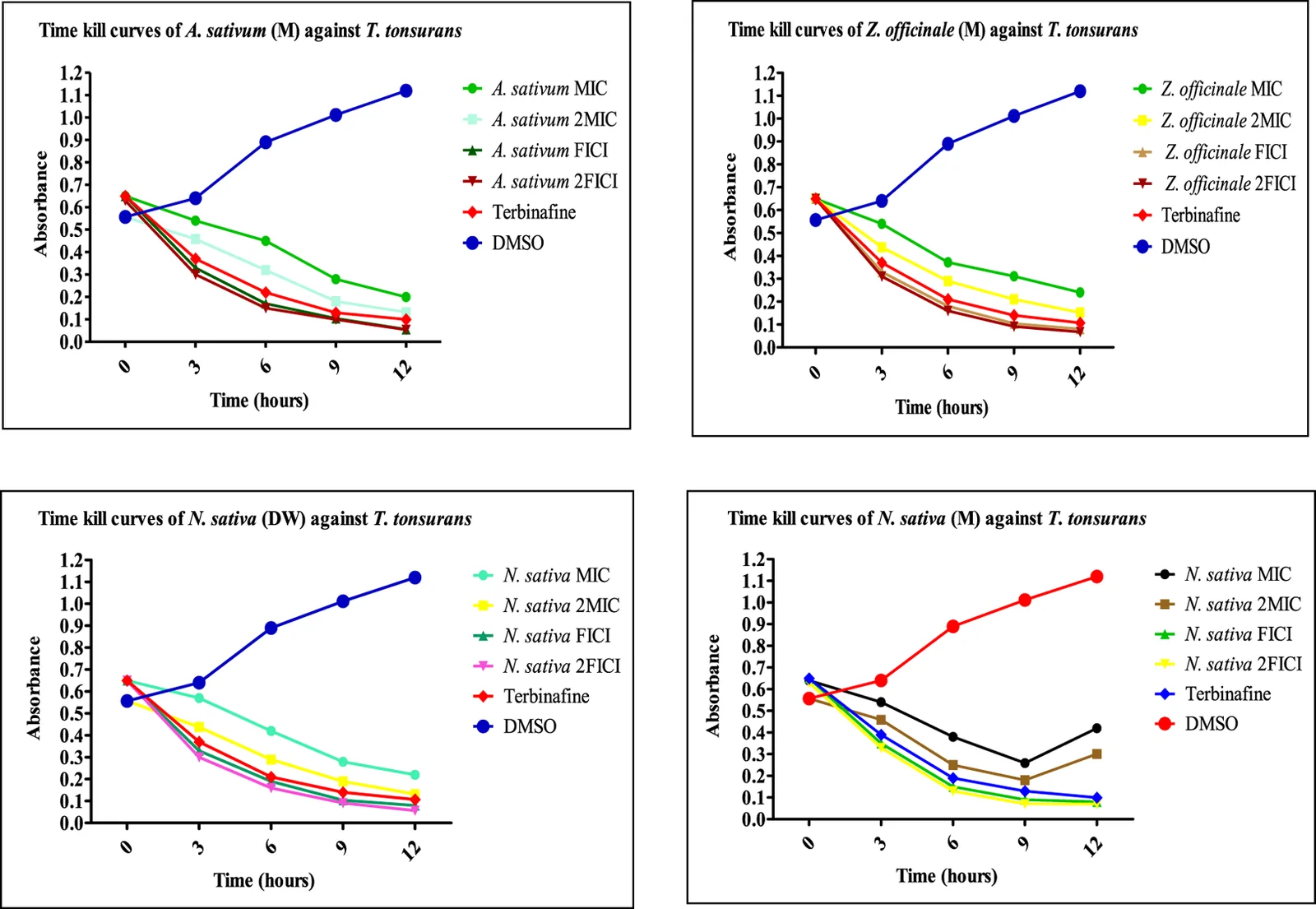
Time-kill curves of selected extracts against dermatophytes. M, methanolic extract; DW, distilled water extract.

**FIGURE 9:**
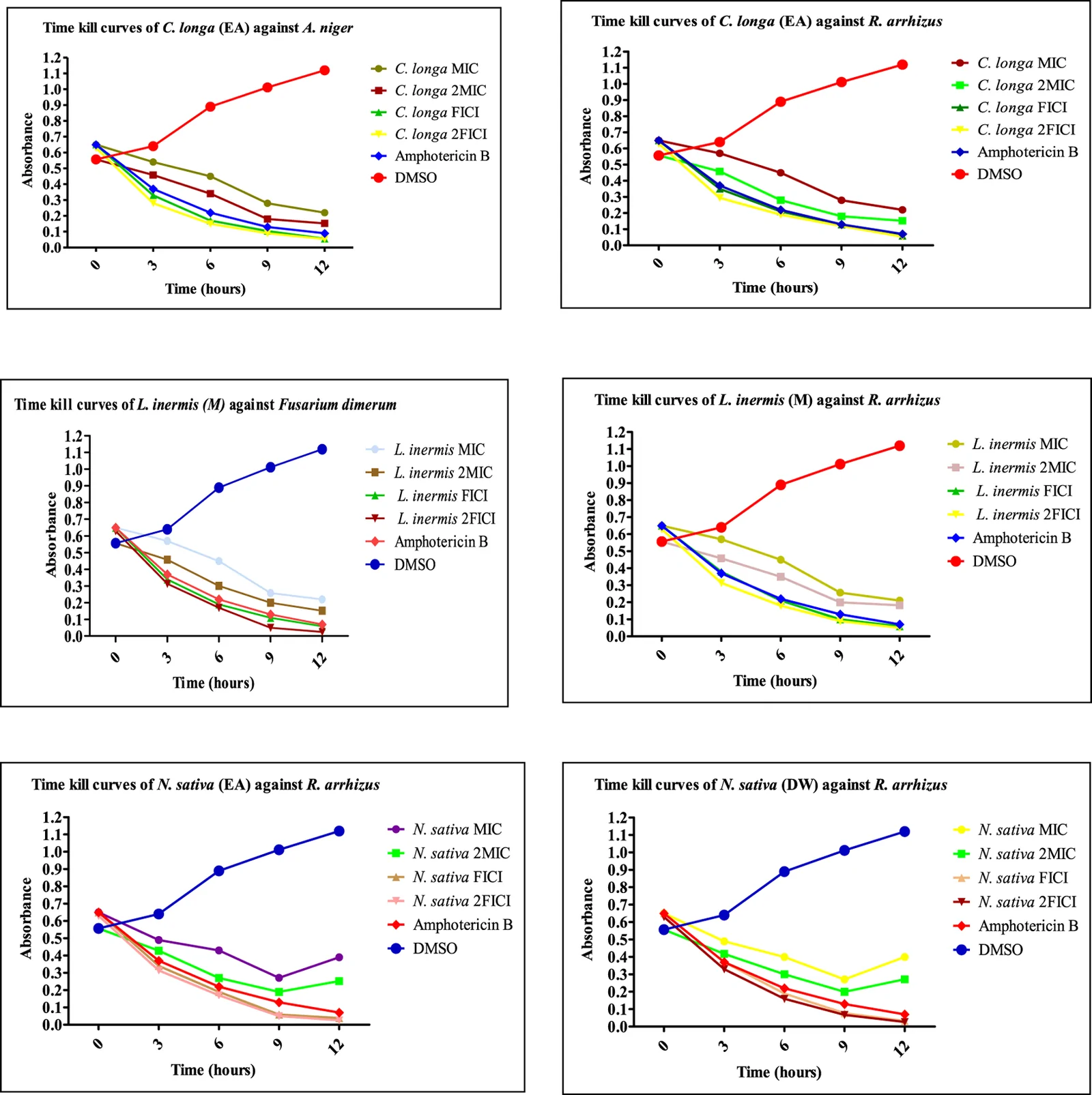
Time-kill curves of selected extracts against non-dermatophytes. EA, ethyl acetate extract; M, methanolic extract; DW, distilled water extract.

The extracts showing an 8-fold reduction in MICs of both extracts and antifungal drugs were further evaluated for their antifungal action up to 12 h, and the results were expressed as a plot showing the time-kill kinetics. Time-kill curves of *Z. officinale, L. inermis, A. sativum,* and *N. sativa* against *T. rubrum and T. tonsurans* showed decline in the fungal growth throughout the increasing time when treated with extracts alone and in combination with terbinafine/AMB, and the time-kill curves of *N. sativa* (M) and *M. piperita* (DW) extracts showed decline in fungal growth from 0 to 9 h and then an increase in fungal growth during 9–12th hours when treated against *T. tonsurans* alone, but in combination with terbinafine/AMB, they showed a time-dependent decrease in fungal growth. For non-dermatophytes, *C. longa* (EA) and *L. inermis* (M) extracts when treated against *R. arrhizus* showed no significant decrease in fungal growth from 9 to 12 hours, but when treated in combination with drugs, they exhibited increased killing of fungi with increasing time; however, *N. sativa* extracts showed increased growth from 9 to 12th hour when treated alone. Figures 7–9 display the time-kill curves of extracts with both dermatophytes (*T. rubrum* and *T. tonsurans*) and non-dermatophytes (*A. niger*, *R. arrhizus,* and *F. dimerum*). The time kill assay provides us with important information, such as time-dependent or concentration-dependent anti-fungal activity. Each sample is targeted at four different concentrations, such as MIC, 2MIC, FICI, and 2FICI. The time-kill assay produces more accurate data concerning the effect of the combinations since the measurements are taken over time (Adusei et al., 2019; Cai et al., 2022). The time-kill curves in this study give us an idea of the possible mechanism of action of our tested extracts when treated alone, and when treated in combination, the continuous decline in fungal activity with increasing time depicts that the reference drugs and tested extracts have acted in synergism.

### 4.7 Protein estimation

A standard curve of BSA was established between the absorbance and different concentrations of BSA to estimate the unknown concentration of protein in fungi-treated samples at their MIC and FICI, i.e., y = 0.0104x − 0.0016 (*R* ^2^ = 0.9992). Table 7 shows the protein concentration and percent protein inhibition of the selected extracts when treated alone and in combination with commercial drugs against the selected dermatophytes and non-dermatophyte strains. It is very evident from the results that the percent protein inhibition increased when the extracts were combined with antifungals. In the case of *T. rubrum,* the *Z. officinale* (DW) extract showed the highest protein inhibition of 92%. For *T. tonsurans,* the *A. sativum* (M) extract showed 80% inhibition. In the case of non-dermatophytes, the *L. inermis* (M) extract showed 77% protein inhibition of *Rhizopus arrhizus,* whereas the *C. longa* (EA) in the case of *Aspergillus niger* and the *L. inermis* (M) extract for *Fusarium dimerum* showed 64 and 52% inhibition of protein, respectively, when combined with AMB. Disintegration of the cell membrane was measured by quantifying the leakage of cellular proteins due to cell death. Protein content in the extracellular medium of treated and untreated fungal strains was analyzed to understand the primary cause of antifungal effect. Standard BSA curves were used to calculate the unknown protein concentration leaked into the medium with or without exposure to test compounds at their MIC and FICI. It is already indicated in the literature that AMB binds with the ergosterol of the cell membrane causing impermeability to proteins and eventually causing cell death, thus acting as fungicidal agents (Cavassin et al., 2021). Similarly, when the tested extracts and AMB were given in combination, there was an increase in percent protein inhibition; thus, we can say that both acted in synergism, possibly by altering membrane permeability. In the same way, terbinafine acts as fungicidal agent by inhibiting squalene epoxide and hindering the biosynthesis of ergosterol, so the cell membrane becomes deficient in ergosterol. The fungicidal action is closely associated with the development of high intracellular squalene concentrations, which are believed to interfere with fungal membrane function and cell wall synthesis (Paskiabi et al., 2017; Wang et al., 2018). It is also evident that when combined with extracts, the concentration of proteins was lower than that of the extracts alone. Therefore, it would not be wrong to say that both extracts and terbinafine interfere with fungal cell walls and might be acting by disrupting the proteins of the fungi, but the mode of action by which they might have acted is not clear. The observed synergistic activity may be attributed to the presence of phytochemicals with documented antifungal activity by interfering with the cell wall, as described before, e.g., catechin (Yang and Jiang, 2015), gallic acid (Li et al., 2017), quercetin (Bitencourt et al., 2013), rutin, and kaempferol (Bae et al., 1997; Reddy et al., 2016), and therefore it can be inferred that when these polyphenols were combined with AMB and terbinafine, they acted in synergy (Wang et al., 2017). Thus, it is evident from the results that these extracts prove to be very useful, which leads in the treatment of onychomycosis. Since these plants are readily available and have already been used as condiments and have been used in the past for many other diseases, there are much less safety and efficacy concerns as compared to other drugs, and because this study was conducted on herbal extracts, these can be used as herbal remedies against onychomycosis. Time-kill kinetics and protein content estimation for the aforementioned synergistic combination have also been conducted for the very first time in this study.

**TABLE 7:** Percent protein inhibition of extracts treated with dermatophytes and non-dermatophytes.

Treated samples	MIC or FICI (μg/ml)	Concentration of protein in μg	Percent protein inhibition
*Trichophyton rubrum*			
*Z. officinale (M)*	31.25	63	6
*Z. officinale (M)*	31.25	55	18
Z. O(M) +terbinafine	3.9 + 0.00625	20	70
Z. O (DW)+terbinafine	3.9 + 0.00625	5	92
Terbinafine	0.005	11	83
Control	--	67	--
*Trichophyton tonsurans*			
*A. sativum* (M)	31.25	45	41
*Z. officinale* (M)	31.25	66	14
*M. piperita* (DW)	31.25	47	38
*N. sativa* (M)	31.25	37	52
*N. sativa* (DW)	31.25	33	57
*L. inermis* (M)	31.25	41	47
*L. inermis* (DW)	31.25	22	72
A. S(M)+ terbinafine	3.9 + 0.00625	15	80
Z. O(M) +terbinafine	3.9 + 0.00625	22	71
M. P (DW)+terbinafine	3.9 + 0.00625	33	57
N. S (M) +terbinafine	3.9 + 0.00625	24	69
N. S (DW) +terbinafine	3.9 + 0.00625	19	75
L. I(M) +terbinafine	3.9 + 0.00625	24	69
L. I(M) +terbinafine	3.9 + 0.00625	29	57
Terbinafine	0.005	13	83
Control	--	77	--
*Rhizopus arrhizus*			
*N. sativa* (EA)	31.25	44	6
*N. sativa* (DW)	31.25	23	51
*L. inermis* (M)	31.25	39	17
*C. longa* (EA)	31.25	29	38
N. S (EA)+ Amphotericin B	3.9 + 0.4	31	34
N. S (DW) +Amphotericin B	3.9 + 0.4	24	49
L. I(M) + Amphotericin B	3.9 + 0.4	11	77
C. L (EA) + Amphotericin B	3.9 + 0.4	19	59
Amphotericin B	3.2	6	87
Control	--	47	--
*Aspergillus niger*			
*C. longa* (EA)	31.25	35	57
C. L (EA) + Amphotericin B	3.9 + 0.4	29	64
Amphotericin B	3.2	15	81
Control	--	81	--
*Fusarium dimerum*			
*L. inermis* (M)	31.25	38	39
L.I(M) + Amphotericin B	3.9 + 0.4	30	52
Amphotericin B	3.2	8	87
Control	--	62	--

“--”, not observed; control, untreated fungal protein concentration; MIC, minimum inhibitory concentration; FICI, fractional inhibitory concentration index; A. S, *Allium sativum*; Z. O, *Zingiber officinale*; N. S, *Nigella sativa*; M. P, *Mentha piperita*; L. I, *Lawsonia inermis*; C. L, *Curcuma longa*; EA, ethyl acetate extract; DW, distilled water extract; M, methanolic extract.

## 5 Conclusion

The findings of this study suggest that substantial antioxidant potential and the presence of therapeutically significant polyphenols in the extracts of *Azadirachta indica*, *Lawsonia inermis*, and *Zingiber officinale* are indicative of the antifungal activity exhibited by these extracts*.* The determination of antifungal activity confirmed the potential of the extracts of *Allium sativum*, *Nigella sativa*, *Curcuma longa*, and *Mentha piperita* along with the aforementioned extracts, and the combination studies prove that when given together with contemporary antifungals, these extracts can act in synergism and potentiate the antifungal effect of each other. The time-kill kinetics of this study gave an idea toward the possible mechanism of action through time-dependent killing of fungi by these extracts and their combination with contemporary antifungals, while the results of protein estimation provided an indication that disruption of the cell wall might be the cause of antifungal action. All these results point to the fact that these extracts could be used as alternative medicines against onychomycosis.

The present study calls for additional research directed toward isolating the bioactive compounds liable for the detected activity. These compounds may possibly serve as novel scaffolds in the search for new drugs. Further studies involving preclinical *in vivo* studies to confirm the *in vitro* results of these crude extracts are suggested. This research also recommends the development of nanoparticles and metal complexes.

## Data Availability

The original contributions presented in the study are included in the article/Supplementary Material; further inquiries can be directed to the corresponding authors.

## Footnotes

1 DPPH, 2,2-diphenyl-1-picrylhydrazyl; DW, distilled water; EA, ethyl acetate; M, MeOH, methanol; FRSA, free radical scavenging assay; RP-HPLC, reverse phase high-performance liquid chromatography; TAC; total antioxidant capacity; TRP, total reducing power; TPC, total phenolic content; TFC, total flavonoid content; MIC, minimum inhibitory concentration; AMB, amphotericin-B; TCA, trichloroacetic acid; DMSO, dimethyl sulfoxide; FC reagent, Folin–Ciocalteu reagent; SDA, Sabouraud dextrose agar; RPMI, Roswell Park Memorial Institute Medium; ZOI, zone of inhibition; FICI, fractional inhibitory concentration index; AST, antifungal susceptibility testing.
