# Correction: Appraisal of selected ethnomedicinal plants as alternative therapies against onychomycosis: evaluation of synergy and time-kill kinetics

**DOI:** 10.3389/fphar.2026.1956892

**Published:** 2026-09-01

**Authors:** Syeda Aroosa Mohsin, Shazia Shaukat, Marya Nawaz, Tofeeq Ur-Rehman, Nadeem Irshad, Muhammad Majid, Syed Shams ul Hassan, Simona Bungau, Humaira Fatima

**Affiliations:** 1 Department of Pharmacy, Faculty of Biological Sciences, Quaid-i-Azam University, Islamabad, Pakistan; 2 Department of Pathology, Shifa College of Medicine, Islamabad, Pakistan; 3 Faculty of Pharmacy, Hamdard University, Islamabad, Pakistan; 4 Shanghai Key Laboratory for Molecular Engineering of Chiral Drugs, School of Pharmacy, Shanghai Jiao Tong University, Shanghai, China; 5 Department of Natural Product Chemistry, School of Pharmacy, Shanghai Jiao Tong University, Shanghai, China; 6 Department of Pharmacy, Faculty of Medicine and Pharmacy, University of Oradea, Oradea, Romania

**Keywords:** onychomycosis, antifungal, resistance, synergistic studies, HPLC

The reference for Xu et al., 2021 was erroneously written as Xu, S., Tao, H., Cao, W., Cao, L., Lin, Y., Zhao, S.-M., et al. (2021). Ketogenic diets inhibit mitochondrial biogenesis and induce cardiac fibrosis. Signal Transduct. Target. Ther. 6, 54. doi: 10.1038/s41392-020-00411-4. It should be Xu, Y., Zhao, H., Zhang, M., Li, C.J., Lin, X.Z., Sheng, J., et al. (2011). Variations of antioxidant properties and NO scavenging abilities during fermentation of tea. Int. J. Mol. Sci. 12 (7), 4574–4590. doi: 10.3390/ijms12074574.

The original version of this article has been updated.

